# Dependence of Heat Transport in Solids on Length-Scale, Pressure, and Temperature: Implications for Mechanisms and Thermodynamics

**DOI:** 10.3390/ma14020449

**Published:** 2021-01-18

**Authors:** Anne M. Hofmeister

**Affiliations:** Department of Earth and Planetary Science, Washington University, St. Louis, MO 63130, USA; hofmeist@wustl.edu; Tel.: +1-314-935-7440

**Keywords:** laser flash analysis, heat, transport properties, temperature, pressure, length-scale physics, optical thickness, radiative diffusion, infrared absorption

## Abstract

Accurate laser-flash measurements of thermal diffusivity (*D*) of diverse bulk solids at moderate temperature (*T*), with thickness *L* of ~0.03 to 10 mm, reveal that *D*(*T*) = *D*_∞_(*T*)[1 − exp(−*bL*)]. When *L* is several mm, *D*_∞_(*T*) = F*T*^−G^ + H*T*, where F is constant, G is ~1 or 0, and H (for insulators) is ~0.001. The attenuation parameter *b* = 6.19*D*_∞_^−0.477^ at 298 K for electrical insulators, elements, and alloys. Dimensional analysis confirms that *D* → 0 as *L* → 0, which is consistent with heat diffusion, requiring a medium. Thermal conductivity (*κ*) behaves similarly, being proportional to *D*. Attenuation describing heat conduction signifies that light is the diffusing entity in solids. A radiative transfer model with 1 free parameter that represents a simplified absorption coefficient describes the complex form for *κ*(*T*) of solids, including its strong peak at cryogenic temperatures. Three parameters describe *κ* with a secondary peak and/or a high-*T* increase. The strong length dependence and experimental difficulties in diamond anvil studies have yielded problematic transport properties. Reliable low-pressure data on diverse thick samples reveal a new thermodynamic formula for specific heat (∂ln(*c_P_*)/∂*P* = −linear compressibility), which leads to ∂ln(*κ*)/∂*P* = linear compressibility + ∂lnα/∂*P*, where α is thermal expansivity. These formulae support that heat conduction in solids equals diffusion of light down the thermal gradient, since changing *P* alters the space occupied by matter, but not by light.

## 1. Introduction

The transport of heat accompanies disequilibrium conditions. Thus, heat transport properties differ fundamentally from thermodynamic properties, which describe behavior independent of time. Understanding the dynamical process of heat flow is difficult because temperature is generally monitored, which differs from heat. In addition, heat transport data on electrical insulators are commonly impacted by two systematic errors with opposite signs: ballistic (boundary-to-boundary) radiative transfer augmentation and thermal losses at physical contacts with thermocouples and heaters.

These effects are not small. For hard oxides against metals, losses are ~10% per contact [1]. However, spurious radiative transport is the greater problem. This artifact overwhelms conduction at high temperatures (*T*) due to blackbody radiation intensity increasing as *T*^4^ [2]. The high-temperature transmission of visible light through a sample is apparent to an experimenter, but not so obvious is low-*T* passage of near-infrared (IR) light, the frequencies of which (ν) are relevant below ~500 K. Nonetheless, such artifacts have been recognized in steady-state cryogenic experiments [3].

Spurious radiative processes depend on scale-length, as implied by the designation of boundary-to-boundary transfer. In detail, the amount of light exiting the sample is measured, but the measured transmission (*I_trns_*) is impacted by surface reflectivity (*r*). For normal incidence at intensity *I*_0_ and parallel light rays crossing a flat slab of thickness *L*, the absorption coefficient (*A*) is:

(1) A(ν)L=−ln[Itrns(ν)I0(ν)]+2ln[1−r(ν)]

From Equation (1), spurious augmentation during heat transfer depends on not only temperature and spectral characteristics, but moreover on the experimental conditions of length-scale and interface reflections. The narrow range of sizes (~1 to 5 mm) used in most transport studies of bulk material limits variation in the amount of ballistic transfer [4] (Chapter 4). Since *L* has not been considered as relevant, and is not always reported, this systematic error mimics a random error. Uncertainties in any given measurement from these two compensating systematic errors combined are modest near 298 K, rendering the data useful for many applications. However, delineating microscopic behavior requires accurate methods which isolate the process of interest.

Thin film studies confirm that isolation is needed. For bulk samples, high and low frequency ranges, respectively, represent ballistic and diffusive mechanisms. However, ballistic transport can exist in the infrared across samples ~20 to ~1000 nm thick (e.g., [5,6,7]). This special process involves reflections from interfaces and minimal interactions with heat as it travels across the sample.

Regarding the interactive process of heat diffusion, currently, popular microscopic models are derived from the elastic kinetic theory of gas (EKTG). Significant problems exist: (i) Elastic collisions are assumed [8]. However, under this condition, temperature cannot evolve. EKTG rests on data on gases that were collected under negligible thermal gradients, needed to avoid convection. The resulting nearly isothermal conditions provided insignificant heat transfer, and so reasonable agreement was obtained [4]. (ii) A factor of 3 error exists in all such models due to assuming that heat is scattered in all directions, which does not describe heat flowing down the temperature gradient, as required by thermodynamic law [9]. (iii) In solids, heat and mass move independently, but in gas, heat and mass move inseparably. Hence, EKTG is inconsistent with the form of Fourier’s time-dependent heat equation differing from Fick’s (Section 2), and so cannot represent dynamic behavior.

To better understand heat transport in solids, a large database was constructed on thermal diffusivity (*D*) above ~298 K on ~200 non-metallic crystals, ~100 silicate glasses, and ~30 metals using laser-flash analysis (LFA) [4,9,10]. This contact-free technique was developed in 1961 by Parker et al. [11]. Subsequent developments removed the remaining systematic error of ballistic radiative transfer [12,13,14,15,16], making LFA the most accurate modern method (others are ±5% [17]). Measuring diverse materials has shown that the diffusion of low-frequency IR light transports some heat in electrical insulators [11] and that a vibrational mechanism is important to metals and alloys [9]. Radiative diffusion merits further investigation because the popular mechanism of phonon–phonon scattering has shortcomings beyond the three listed above. (iv) Phonons are pseudo-particles, unlike the photon which is real. (v) Photons are heat-energy and are everywhere. (vi) Photons readily leave a body during heat transfer, but not electrons or phonons.

### 1.1. Summary of the LFA Database

Thermal diffusivity data were collected above 298 K on (i) elements and alloys; (ii) diatomic compounds (oxides, halides, PbS and SiC); (iii) XF_2_ phases; (iv) chemically and structurally simple oxides with minor cation disorder (perovskite, corundum, and spinel); (v) calcite and other carbonates; (vi) structurally complex, end-member silicate minerals (quartz, forsterite, albite); (vii) silicates with high cation substitution (garnet, olivine, pyroxene, phyllosilicates, zeolite); (viii) disordered silicates (glasses, framework structural classes). The focus is thus on silicates and oxides. For summaries, see [9,10,18] and [4] (Chapters 7, 9, 10).

Because not all LFA studies remove spurious radiative transfer (e.g., ceramics, where the effect is weak), data from others are utilized only when it is certain that ballistic effects are absent.

### 1.2. Purpose and Organization of the Paper

To better delineate the role of photons in diffusing heat, this paper extends prior exploration of the length dependence of *D* in bulk materials to include metals, and evaluates the pressure dependence of *κ*, since light and matter react much differently to compression. Section 2 discusses macroscopic heat transfer theory. Section 3 covers the LFA method, including how ballistic radiative effects are addressed. Section 4 provides results at ambient pressure (*P*), quantifying the length and temperature dependence of *D* for bulk solids with diverse bonding types, structures, and chemical compositions. Section 5 covers issues in high-pressure diamond anvil cell studies, and extracts trends from accurate data collected mostly in piston-cylinder devices, leading to new connections of transport with thermodynamic properties. Section 6 compares a model for diffusion of radiation with data. Section 7 summarizes.

## 2. Macroscopic Theory of Heat Diffusion

By 1822, Fourier had constructed today’s model for conduction by visualizing heat flowing in rays through the space occupied by a body. Fourier assumed that small internal elements of the body stored heat whereby the amount of heat transferred between pairs of elements is proportional to their temperature difference [19]. Modern expressions of flux (ℑ = energy per area per time) and the defining linearized equation for variable thermal conductivity (*κ*) are:

(2) $$ℑ=-\kappa \nabla T\;\mathrm{and}\;\rho c_{P}\frac{\partial T}{\partial t}=\nabla •\kappa \nabla T$$

where *t* is time, *ρ* is density, and *c_P_* is specific heat at constant pressure *P* (heat per unit mass per degree). Although constant strain pertains, constant *P* suffices [20]. Utilizing energy conservation yields the right-hand side (RHS) from the left (LHS). Fourier’s equations describe the diffusion of heat.

If temperature changes are sufficiently small that *κ* varies insignificantly, (2) reduces to Fourier’s original heat equation:

(3) $$\frac{\partial T}{\partial t}=D\nabla^{2}T;\;\mathrm{in}\;1-\mathrm{dimension}:\;\frac{\partial T}{\partial t}=D\frac{\partial^{2}T}{\partial z^{2}}$$

where thermal diffusivity and *κ* are linked via:

(4) $$\kappa =\rho c_{P}D\equiv CD$$

### 2.1. Lumped Parameters

Transtrum et al. [21] showed that ambiguous behavior describes models which contain lumped (multiplied) factors. Per Equation (4), trade-offs accompany measurements of *κ*. Since density is part of storativity (*C* = *ρc_P_*), the quantity of which directly links heat content to space, then from Equation (4), large *κ* could represent a lot of heat moving quite slowly, or a small amount of heat moving very fast. To understand the transport process, information on thermal diffusivity and heat capacity are essential, as *D* governs how fast thermal fields change, while *C* indicates how much heat is moved.

### 2.2. Dimensional Analysis of Fourier’s Equation

Because heat flows down a temperature gradient, the one-dimensional Cartesian form of Equation (3) embodies the physics of heat diffusion. All solutions to the RHS of Equation (3) have the form:

(5) D~L2ζ

where *L* is the length across which heat diffuses and *ζ* is a thermalization time. The gradual warming of a body with time can be represented as the motion of a heat front. Linearizing the speed across the sample gives *u* ~ *L*/ζ, and thus:

(6) u~DL or D~uL.

As heat is conducted across a sample with some particular *L*, mechanisms with substantially different *D* have cooling fronts that progress at substantially different speeds. These fronts can be distinguished in transient experiments, such as LFA, where *T* is measured as a function of *t*, but not in periodic experiments, which average measurements over some time interval [4] (Chapter 4). Measuring the thermal evolution after a heat perturbation sheds quantitative information on the rate and mechanism by which energy diffuses through a material [9].

If only one process exists, and experiments are conducted by varying *L* at constant *T*, then the dependence of *D* on *L* is revealed. Equation (6) suggests a roughly linear dependence. The limit is:

(7) D→0 as L→0

Equations (5) and (6) and the consequent limiting values of Equation (7) are consistent with diffusion requiring a participating medium. Ballistic transfer, in contrast, negligibly involves the medium being traversed, yet is influenced by length, since transmission never reaches 100%, as per Equation (1).

Dimensional analysis shows that the distance over which heat diffuses must be independently known to quantify *D*, and thus *κ*. Although this inherent limitation seems to have been overcome via techniques involving extensive numerical modelling, in practice such approaches rely on benchmarking against previous experiments [4,17], which almost always used large, mm sized samples. Experiments (Section 4) verify that the length-scale dependence exists in bulk samples.

### 2.3. The Importance of Space and Heat Capacity

Time-dependent diffusive flow of *matter* (Fick’s equations) at any given *T* involves only mass and distance because concentration links mass to space occupied. In contrast, time-dependent diffusive flow of heat (Equation (2)) involves energy, distance, and temperature, because the amount of heat depends on two independent variables: volume (*V*) and temperature. For this reason, one more parameter, *c_P_*, appears in Fourier’s equations, describing the flow of energy, in comparison to in Fick’s equations for the flow of mass. Storativity (*C* = *ρc_P_*) is important during heat transport due to tradeoffs in Equation (4) and, moreover, because it links temperature with heat and space:

(8) $$\Delta T≅\;\frac{Q}{aLC}=\frac{Q}{CV}$$

where *Q* is the heat applied and *a* is area [11].

**Figure 1:**
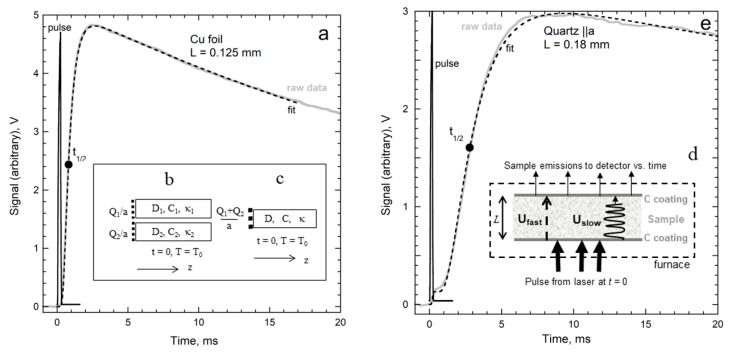
Thermal evolution in LFA (raw data) for thin samples: (**a**) Temperature-time curve of copper foil at 298 K, which initially heats due to the laser pulse (solid curve) and then cools to the surroundings. Raw data (grey) are fit with Cowan’s model. Black dot = position of *t*_½_. (**b**) Schematic of initial conditions in two parallel bars. Heavy dots represent application of a pulse. (**c**) Schematic of initial conditions for a blended bar, where *Q* = *Q*_1_ + *Q*_2_. (**d**) Operation essentials of LFA. Dashed box indicates the furnace enclosing the sample. Speckled rectangle depicts the edge-on the sample of thickness *L.* Grey shows graphite coatings. Arrows indicate arrival of laser energy and departure of emissions. Dashed arrows show fast ballistic transfer. Squiggle arrows indicate slow diffusive travel of heat across the sample. (**e**) Temperature–time curve of quartz at 298 K, showing a small amount of ballistic transfer, addressed by modeling (Section 3.1.2). Schematics after Figure 5a and Figure 21a,b from Criss and Hofmeister [9], which is open access.

Equation (8) presumes that a single temperature field applies. To delineate parallel flow, Section 2.4 uses (8) and an equivalent conductivity (*κ*_equivalent_), which is an approximate value that conserves energy while representing thermal evolution as a simple continuous rise in *T* with time as the heat traverses the sample (Figure 1a). Section 3 covers disequilibrium conditions in experiments immediately after a heat pulse is applied, at which time the very fast carriers (photons or electrons) may be traveling.

### 2.4. Parallel Flow Depicts Co-Existing Mechanisms in Condensed Matter

Parallel heat flow involving simultaneous, but independent mechanisms, is described by multiple bars of equal density, wherein only one mechanism operates in each bar (Figure 1b,c). Temperature fields differ in the bars at disequilibrium, which immediately follows a thermal disturbance. Criss and Hofmeister [9] derived sum rules for parallel bars via an adiabatic approximation, summarized as follows:

Conserving energy requires that the apparent temperature field is a weighted sum of temperature fields associated with each mechanism in the differing bars:

(9) $$CT=C_{1}T_{1}+C_{2}T_{2}=\sum C_{i}T_{i}$$

Assuming that the independent mechanisms each obey Fourier’s law leads to:

(10) $$\kappa \frac{\partial T}{\partial x}=\kappa_{1}\frac{\partial T_{1}}{\partial x_{1}}+\kappa_{2}\frac{\partial T_{2}}{\partial x_{2}}+\kappa_{n}\frac{\partial T_{n}}{\partial x_{n}}=\sum \kappa_{i}\frac{\partial T_{i}}{\partial x_{i}}\;$$

where the subscripts indicate which bar each mechanism operates in. From [9], combining Equations (9) and (10) gives:

(11) $$\kappa \sum C_{i}\frac{\partial T_{i}}{\partial x_{i}}=C\sum \kappa_{i}\frac{\partial T_{i}}{\partial x_{i}}\;$$

Solving Equation (11) requires additional assumptions. A few limiting cases describe situations generally encountered, as follows:

For *n* independent mechanisms, *C* = Σ*C_i_*. If bar properties are similar, then temperature rises are similar, leading to:

(12) $$\kappa =n\frac{\sum C_{i}\kappa_{i}}{\sum C_{i}}\;\mathrm{for}\;C_{i}\approx C_{j}\;\mathrm{and}\;D_{i}\approx D_{j}\;$$

When similar *C_i_* and *D_i_* values exist, the *T* vs. *t* curve represents the average behavior of the various carriers.

If the *D* values vary so that the temperature fields vary relative to one another, then:

(13) $$\kappa ≅\sum \kappa_{i}C_{i}\frac{\sum C_{i}}{\sum C_{i}^{2}}\;$$

To simplify Equation (13), we consider two mechanisms where the specific heats of each mechanism differ significantly, i.e., *C*_1_ << *C*_2_ ≈ *C*. If *κ*_1_ is either > or ~*κ*_2_, then *D*_1_ >> *D*_2_. Such disparate thermal diffusivity values result in great differences between the time evolution of *T* with distance associated with each process. Over a relatively short time, fast process 1 will equilibrate over the whole bar. Thus, bar 1 representing process 1 soon becomes isothermal, so *∂T*_1_/*∂z*_1_ = 0. In contrast, over this same short time interval, *T*_2_ in bar 2 is still changing with distance, providing a heat current of *κ*_2_∂*T*_2_/∂*z*_2_. Summing currents gives *κ∂T*/∂*z* = *κ*_2_*∂T*_2_/*∂z*_2_ + 0 and thus:

(14) $$\kappa =\kappa_{2},\;\mathrm{for}\;C_{1}<<C_{2}.$$

For completeness, we now assume that *κ*_1_ < *κ*_2_, while keeping *C*_1_ << *C*_2_. Hence, mechanism 1 has a negligible effect on heat transfer, so Equation (14) again holds.

Importantly, the weighted sum of Equation (13) reduces to Equation (14) if *C*_1_ << *C*_2_, showing that Equation (13) is more general than as suggested by the conditions of the derivation. Regarding Equation (12), this implicitly assumes that the temperature fields are nearly same, and thus describes a single mechanism with multiple carriers (e.g., vibrational transport).

Storativity (*C* = *ρc_P_*) is equally as important a parameter as thermal conductivity in describing heat transfer in a material with multiple mechanisms. Consequently, the slower process dominates, should it have a significantly higher *C*. Equation (14) describes metals, as electrons (mechanism 1) carry negligible heat compared to vibrations (mechanism 2). Tradeoffs in the product of (4), the factor of 3 error in ETKG, analogies to electrical currents only holding near equilibrium conditions (per Maxwell, since charge is conserved but not heat), and the focus on conventional methods, which describe heat flow long after a disturbance occurred, underlie the acceptance of electrons transporting heat in metals [9,22], despite recognized shortcomings [23,24]; see Section 3.1.3.

## 3. The Method of Laser Flash Analysis

The advantages of the transient LFA technique [11,25] include avoiding physical contacts, which inhibit flow. Because length and time, not heat input nor absolute temperature, are quantified, accuracy (±2%) is a factor of two better than in other techniques [17]. The many publications on LFA and continued developments, including the removal of spurious radiative transfer, demonstrate utility and versatility, e.g., [26,27].

Because this study uses LFA data and because some DAC experiments will be evaluated using LFA models, this section provides details. One goal is to clarify how applying an intense heat pulse of short duration differs from conditions in LFA.

### 3.1. Principles and Essentials

The outcome of an experiment depends on how the heat applied depends on time, per Fourier’s equations. In LFA experiments, a flat slab is held at some temperature independently, and then its surface is heated incrementally and remotely by a short, high power light pulse from a laser or UV lamp (Figure 1d). The temperature increase generated by the light pulse propagates though the well-defined thickness (*L*) of the slab. Temperature is ascertained by recording rear-surface emissions remotely with an IR detector. As long as the amount of applied heat (*Q*) applied is small, the temperature rise, as per Equation (8), is small (<4 °C) and the change in emissions is proportional to temperature [26]. Consequently, raw data are denoted as temperature–time (*T*-*t*) curves, and these can be analyzed via Equation (3). To provide high accuracy requires:

- Parallel ray geometry (not spot heating) and flat sample shape so heat flow is one-dimensional.
- A front surface coating (e.g., graphite) to provide a blackbody spectrum which diffuses.
- Small *T* changes from the pulse and a negligible initial thermal gradient existing across the sample, so transport occurs under approximately isothermal conditions.
- A rear surface coating so emissions are collected from the surface, not from the interior.
- A well-defined application time of the pulse and a known length over which diffusion occurs.

An asymmetric “S” shape (Figure 1a) results from the gradual diffusion of the applied heat from the front to rear surfaces, which is accompanied by loss of heat from the rear surface as the sample re-equilibrates. The physics is gleaned from a relationship describing simpler, adiabatic conditions where *T* remains constant and high for a substantial interval after the pulse, as occurs for thick metals slabs [11,25]:

(15) $$D=b_{j}\frac{L^{2}}{t_{j}}=0.138785\frac{L^{2}}{t_{1/2}}$$

where *t*_½_ is most convenient, being the time taken for the rear surface to reach half of the maximum temperature (Figure 1a). Because distance and time are accurately measured, *D* is accurately measured. Importantly, Equation (15) has the same form as Equation (5), obtained from dimensional analysis. Parker et al.’s. [11] adiabatic model thus supports the limits of Equation (7).

#### 3.1.1. Model for External Radiative Cooling

**Figure 2:**
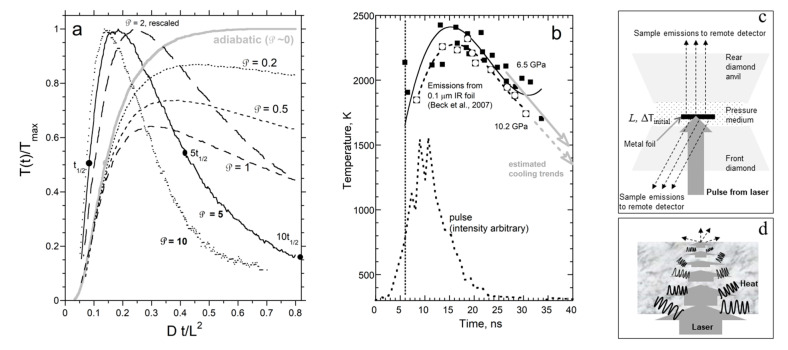
Cowan’s model for LFA contrasted with DAC laser heating experiments: (**a**) Theoretical dimensionless cooling curves that assume small temperature changes and a narrow laser pulse (spike at *Dt*/*L*^2^ = 0) as in LFA experiments. Small radiative losses (℘ < 1) are calculated using Equation (16) without modification, whereas *T*-*t* curves for large losses (℘ = 2, 5, or 10) from (16) are rescaled with maximum temperature set near unity. Scaling provides a visual curve more like LFA data (Figure 1) and better shows the downshifting of the halftime. Dots = *t*_½_ and its multiples. (**b**) Temperatures (squares) ascertained by Beck et al. [30] from fitting emissions from laser heating the rear side of a 0.1 μm thick foil of iridium sandwiched between thin MgO slices in a DAC. Data were digitized and a polynomial fit was made to emissions processed at two different pressures. Unlike LFA, the laser serves as an unsteady furnace that provides a large surge in *T,* rather than adding an increment of heat. (**c**) Schematic of diamond anvil experiments on uncoated metal films. (**d**) Schematic of heat generated as the laser pulse penetrates and attenuates as it crosses an uncoated sample. Part (**b**) is modified after Beck et al. [30] with permission from AIP.

The re-equilibration of the sample with the surroundings after the laser perturbation subsides not only causes *T* to decrease at long times (Figure 1a), but moreover reduces the maximum temperature that can possibly be attained while shifting the maximum to shorter times (Figure 2a). Because changes in *T* are small, losses can be linearized. By assuming adiabatic warming and an initial null temperature (which is consistent with applying a baseline correction to the emissions), Cowan [28,29] devised a model which converges rapidly and closely describes opaque materials:

(16) $$\frac{T(L,t)}{T_{\max}}=\exp\left(\frac{-℘Dt}{L^{2}}\right)+2\sum_{n=1}^{\infty }{\left(-1\right)}^{n}\exp\left(\frac{-n^{2}\pi^{2}Dt}{L^{2}}\right)$$

The cooling parameter (*℘*) is 0 when no radiative loss exists. Cowan approximated *℘* by:

(17) $$℘\approx 2.3\times 10^{5}L\left[1+\frac{c_{x=L}}{c_{x=0}}\right]{\left(\frac{T}{1000}\right)}^{3}\frac{\varepsilon }{\kappa }$$

where emissivity (*ε*) is non-dimensional and the units of *κ* are ergs cm^−1^K^−1^. For small *T* changes, the ratio of the specific heat nearly equals unity.

Each temperature–time curve is modeled by fitting the signal to Equation (16), with ℘ as a constant fitting parameter. Accurate values can be obtained graphically [29] from temperature ratios. Figure 2a indicates the relevant parameters, which are:

(18) T(5t1/2)T(t1/2) or T(10t1/2)T(t1/2)

#### 3.1.2. Model Removing Effects of Fast Internal (Ballistic) Transport from *T-t* Curves

Processes contributing to measured emissions can be distinguished when their characteristic speeds (Equation (6)) differ. Ballistic radiative transfer occurs at roughly *c*/*n*, where *c* is lightspeed and *n* is the index of refraction. Diffusive transfer is much slower due to interaction of heat with the medium (Section 5). Ballistic transfer is thus visually discernable from diffusion in the temperature–time curves, especially when *T* (and thus flux) is high (Figure 1e, see also [31,32,33,34,35]). The ballistic increase occurs over a small time interval because the pulse has finite duration.

Internal radiative processes can be virtually eliminated by applying a thin metal coating [12]. However, coatings with much different *D* than the sample can affect the particulars of heat transfer, so the removal of ballistic transport by a model is highly advantageous.

Blumm et al. [13] devised a formulation assuming optically thin conditions in the near-IR. Absorbance depending on frequency is permissible; however, values of optical properties are not required. Fluxes from decoupled mechanisms add up, so under small temperature changes, Equation (2) becomes:

(19) $$\rho c_{P}\frac{\partial T}{\partial t}=-\left(\frac{\partial {ℑ}_{dif}}{\partial z}+\frac{\partial {ℑ}_{bal}}{\partial z}\right)$$

The ballistic flux ℑ*_bal_* arrives from the bottom surface (*z* = 0), since it does not participate in diffusion (subscript dif). The signal is thus the sum of the temperature rise at the top surface from heat diffusion plus a contribution from the bottom surface, presumed to be a small fraction (*χ*) of the total increase in *T*. Adiabatic solutions for one-dimensional cooling for each contribution provide [13]:

(20) $$T(L,t)=T_{\max}\left[1+2\sum_{n=1}^{\infty }{\left(-1\right)}^{n}\exp\left(\frac{-n^{2}\pi^{2}Dt}{L^{2}}\right)\right]+\chi T_{\max}\left[1+2\sum_{n=1}^{\infty }\exp\left(\frac{-n^{2}\pi^{2}Dt}{L^{2}}\right)\right]$$

In numerically fitting Equation (20) to the signal, *χ* serves as a fitting parameter.

Hahn et al. [14] extended the ballistic model to include how radiative heat losses depend on *T*, as well as the effects of both graphite layers. Hofmann et al. [15] demonstrated that the improved model provided an accuracy of ~1% at high temperature, and that the model requires only approximately diathermic materials. Mehling et al. [16] summarized the improved model, conducted experiments on several glasses, and proved that a graphite coating alone suffices. Figure 1e applies their model to thin quartz.

Although the ballistic model is only strictly valid when the sample is either fully transparent, or strongly scattering, or strongly absorbing [14], only a few samples of the hundreds studied in our laboratory produced *T*-*t* curves that departed from this model. Exceptions consist of materials that not only moderately absorb the thicknesses used but also in the near-IR where the blackbody radiation is significant at the temperatures explored. These are deeply colored rare-earth garnets [31] and spinels with low frequency and weak, broad electronic transitions of Fe^2+^ [36].

#### 3.1.3. Sequential Rises in Metals Show Electronic Transport Is Transient and Carries Little Heat

**Figure 3:**
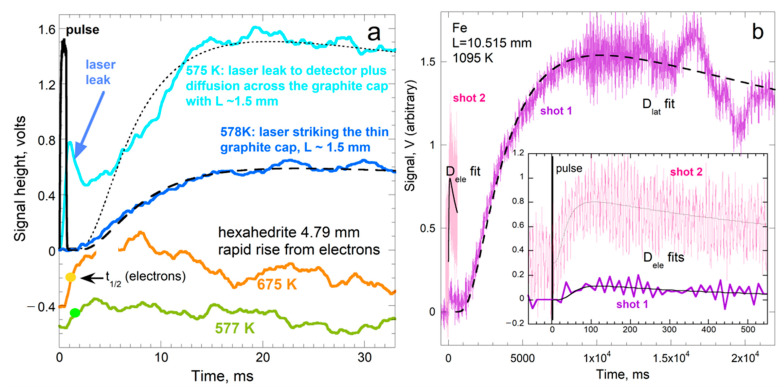
Temperature–time curves revealing fast electronic transport: (**a**) Raw data collected over a short time from a Fe-Ni meteorite with *L* = 4.59 mm at moderate *T*. Blue curves from [9], which is open access, used a holder and cap with apertures permitting laser light to reach the detector and/or to directly heat the thin graphite cap. Orange and green curves, offset for clarity, record emissions only from the meteorite and show electronic heat transport. (**b**) Long sample of electrolytic iron at high *T*. Purple curve shows a long duration where the sample is first heated by electrons, then cools radiatively to the surroundings, and next warms by vibrational transport. Long durations provide instabilities at long times. Both electronic *T*-*t* (inset) and vibrational evolution are fit to Cowan’s model. Pink curve shows data collected over a brief duration. Software used a large gain to meet a 1 Volt minimum signal strength, producing white noise. For long durations, wide spacing of points (inset) also produces noise. Similar *D*_ele_ was obtained in [9] for other metals using short and long durations.

The independent responses of electrons and phonons in metals to fast, intense laser pulses have been demonstrated in fs-spectroscopy [37]. Because carrier speeds for electronic and vibrational mechanisms differ by ×1000, *D*_ele_ should be about 1000 × *D*_lat_, and two independent diffusive mechanisms should be observed in *T*-*t* curves wherein the fast rise is weak, since electrons carry very little heat. Criss and Hofmeister [9] observed a weak signal in *T*-*t* curves for many metals and alloys after the pulse—this behavior is unlike spurious radiative transfer which overlaps the pulse. Additionally, the shape of the rapid rise follows Cowan’s idealized curves (cf. Figure 2a to Figure 3).

Because long lengths and long collection times are required to resolve the rapid weak signal, distinct electronic transport was not recognized earlier. First, using *L* of 1 to 4 mm provides a slow rise and *D* consistent with steady-state measurements [24,38,39], so a rapid signal was not sought. Second, for typical lengths, a rapid rise could resemble ballistic transfer, or be masked by noise, or not be resolved by the time steps. Figure 3 shows that artifacts differ in appearance and properties from those of the brief and weak electronic signature.

Rapid, initial rises exist in metals and alloys when their electronic heat capacity is relatively large [9]. The relatively deep penetration of blackbody emissions (from the graphite coating) also helps stimulate electronic transport. For a brief discussion of electronic and vibrational transport in metals, see [22]. Crucial points that were previously overlooked are:

- Electrons outpacing vibrations means that the electrons enter a “cold” region first, and so can give their heat to the valance electrons (and vibrating cations) but cannot uptake heat from the colder surroundings, due to thermodynamic law.
- Excited conduction electrons have a different set of energy states (levels) than those with ambient temperature, so energy exchange from hot to cold electrons is permitted. One may consider the heat transfer as electrons trading states or as the process involving a transient state.

### 3.2. Methodology: Details of LFA Experiments Utilized in This Report

A laser-flash apparatus consists of a controlled atmosphere furnace, a high-energy pulsed laser (or UV lamp), and an IR detector. Bräuer et al. [40] describe our apparatus.

Samples consist of a small, thin slab with parallel faces (~0.3 to ~15 mm thick by 6 to 15 mm diameter). The slab is held by its edges at some temperature provided by the surrounding furnace and touches neither the thermocouples nor the heater. Curie transitions in metals or melting points are used to calibrate temperature. Top and bottom surfaces of the sample are sprayed with graphite (thickness <1 μm). The bottom coat absorbs the narrow laser pulse while converting it to a broad band blackbody spectrum. The top coat enhances emissions. For details, see [9,31,32,33,34].

For a robust fit to the *T-t* curves, the experimental duration should be ~10 half-times. Because long durations are often accompanied by fluctuations or instabilities, thin samples are used to provide short collection times when ascertaining low *D* (e.g., glasses or disordered silicates). For the contrasting situation of high *D*, the shortest duration available in any given apparatus sets a minimum on *L*. Raw data (Figure 1 and Figure 3) are analyzed as per Section 3.1.1 and Section 3.1.2.

Sources of experimental uncertainty in LFA are exemplified by the adiabatic model (15). The strong dependence of *t*_½_ on thickness (6) makes *L* the main source of uncertainty. Reliability is insured by requiring that measured and model temperature–time curves match for each acquisition. Accuracy for the technique is ~2%, ascertained through benchmarking. Opaque and ductile metals are used as these lack radiative transfer and have good thermal contact, providing accuracy during conventional methods (e.g., [38,39]).

## 4. Measured Thermal Diffusivity at Ambient Pressure

Our early studies applied LFA to typically used ~mm thicknesses and found that systematic behavior for *D*(*T*) exists at 1 atm [9,10]. Section 4.1 summarizes. Subsequent studies of thinner samples showed that the shape of *D*(*T*) depends strongly on *L* for thin non-metals [4] (Chapter 7). Section 4.2 summarizes, presents additional data on electrical insulators, and examines thin metal foils, alloys, and semiconductors. Section 4.3 provides details on *D*(*L*,*T*) for MgO and Yt-stabilized zirconia, which were not discussed in [4]. Importantly, spurious ballistic transport was removed from the raw data.

### 4.1. Results for D(T) at Large L

**Figure 4:**
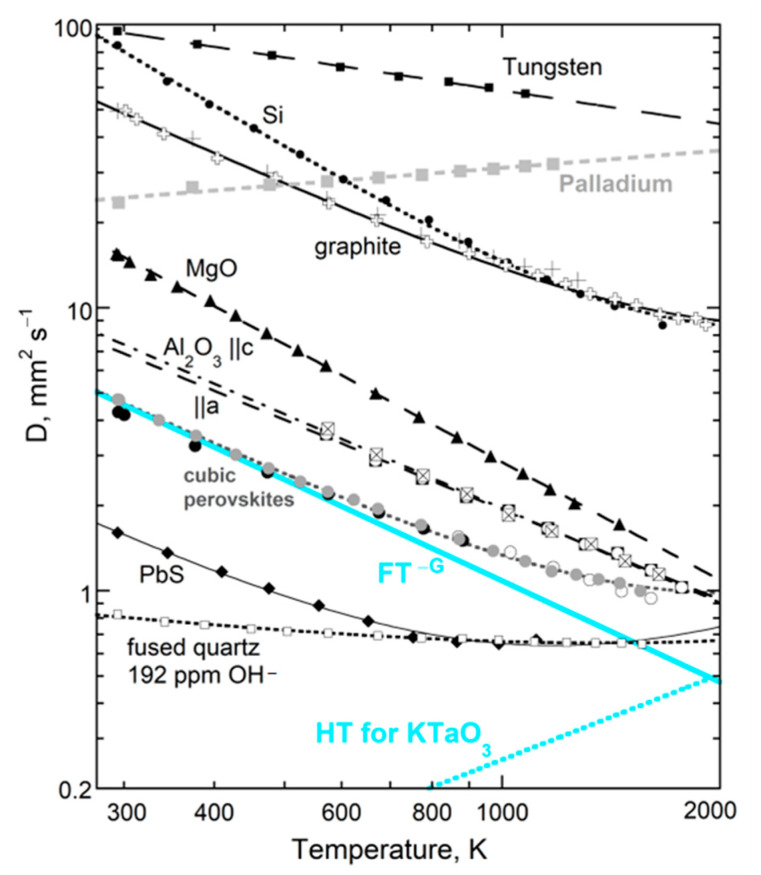
Log-log plot of thermal diffusivity vs. temperature for metals and single-crystal insulators with nearly end-member compositions, as labeled. Structures are various. Thickness, fits, and data sources are shown in Table 1. Because synthetic sapphire is thin, low *T* data points were not used in fitting (see below). Components of the fit for KTaO_3_ perovskite are shown in thick aqua solid and dotted curves.

At ambient conditions, the thermal diffusivity of thick non-metallic samples decreases as *T* increases. The slope is highest near 298 K but progressively decreases, becoming flat near 1000 K. For many electrical insulators, *D*_heat_ at higher *T* increases with *T* (Figure 4). This is intrinsic, since spurious radiative effects were removed. For most elements, *D* decreases with *T*. However, *D* increasing with *T* exists for Pd, Mn as well as many alloys [22] (Table 9.2).

#### 4.1.1. A Universal Law for D(T) at Large L

Over 50 different, large non-metallic single-crystals were fit by a 3-parameter formula [10]:

(21) $$D(T)=FT^{-G}+HT\;\mathrm{or}\;D=F^{*}{\left(\frac{298}{T}\right)}^{-G}+HT$$

where the constants F, G, and H are positive. Figure 4 and Table 1 give examples from [4,10] (Chapter 7). Fits to polycrystals are given by [18]; for glasses, see [41]. For thin samples (*L* < ~1 mm), the form holds above a minimum *T* that exceeds 298 K.

The F*T*^−G^ term describes the strong decrease with *T*, whereas the H*T* term controls the flat region and high *T* upturn in *D*. The dimensions of F depend on G, which can be a non-integer. The alternative form on the RHS of (21) uses F*, which has dimensions similar to *D*. Since H is small, F* = F × (298)^−G^ nearly equals *D*_heat_ at 298 K. Fits were not improved through the use of different powers than those that are linear for the high-*T* response, which signifies radiative transfer [4,10,18].

**Table 1:** Examples of fits ^1^ to thermal diffusivity of nearly pure materials.

Sample	*L*	*D*(*T*)	*T* Range	Source
	mm	mm^2^·s^−1^	K	
W 99.9%	6.35	858.03 *T*^−0.38872^	290–1100	This work
Ti 99.995%	3.64	66.167 *T*^−0.33605^	290–900	[22]
Pd 99.9%	3.45	7.7392 *T*^+0.20185^	290–1200	[22]
Si 99.999%	2.016	54,1490 *T*^−1.5477^ + 0.0022293 *T*	290–1690	[10]
Graphite ZXF-Q5	~2	34,499 *T*^−1.1531^ + 0.0028225 *T*	290–1930	[42]
MgO	0.909	40,667 *T*^−1.385^	290–1460	[43]
Al_2_O_3_ ||c-axis	1.106	4073 *T*^−1.1063^	560–1680	[43]
Al_2_O_3_ ||a-axis	0.993	2835.9 *T*^−1.0555^	560–1770	[43]
KTaO_3_	0.547	3973.9 *T*^−1.1882^ + 0.00025285 *T*	290–1570	[32]
PbS	1.02	1302.4 *T*^−1.1875^ + 0.00029244 *T*	290–1130	[4]
SiO_2_ glass KU2	0.567	3.5582 *T*^−0.2672^ + 9.9943 × 10^−5^ *T*	290–1565	[44]

^1^ Most of the data from previous studies were refit. Linear correlation coefficients are better than 0.99.

Two factors affect whether a finite H*T* term is needed for a good fit of the data to Equation (21): (i) a minimum temperature must be reached, since H is small, and (ii) the structural and chemical complexity of the phase of interest is relevant. Except for PbS, the H*T* term is not needed to describe *D*(*T*) for diatomic compounds, which have simple structures and a single, strong IR band. Galena absorbs into the near-IR range due to its metallic bonding. Single crystals with a diamond structure behave similarly. Although fundamental IR modes are absent, moderately intense impurity bands plus IR overtone/combinations exist. A weak H term is needed to accurately fit Si (Figure 4). Germanium melts below 1000 K, and so H was not constrainable. Data on diamond [4,10] were not sufficiently accurate to detect an H*T* component, due to use of small, inexpensive samples.

In summary, the form for *D*_heat_(*T*) for a material depends on how it absorbs infrared and near-IR light. Complexity increases not only the number of fundamental IR peaks, but more importantly extends their frequency range, which is conducive to diffusive radiative transfer.

#### 4.1.2. Importance of Bond Type to Moderate Temperature Behavior

**Figure 5:**
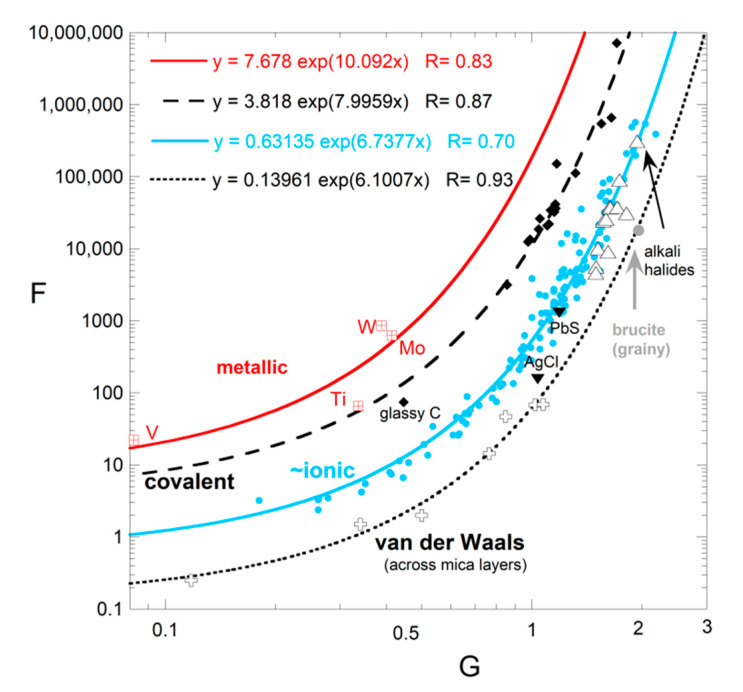
Correlation of fitting coefficients F and G obtained using (21) for all 173 measurements compiled in [4] (Table 7.2) plus metals (Table 1; also [9,11,24,25]). Thicknesses exceed ~1 mm. Fits for anisotropic substances are made to each orientation explored. Exponential fits are provided for the various bonding types. Grainy samples (e.g., brucite, Mg(OH)_2_, and ceramics) fall slightly lower on the curves than similar crystals, due to porosity reducing *D*. Salts lie slightly below the curve for the silicates, which have ionic-covalent bonding. AgCl and metal Ti measurements were over small *T*-ranges, so F and G are less well constrained. Modified after Figure 7.5 in Hofmeister [4] with permissions.

Tradeoffs exist between the coefficients F and G, and to a lesser extent with H, which adds uncertainty to their values. Nonetheless, ranges of parameters are restricted: G ranges from 0.3 to 2, and depends on structure, whereas H is usually near 10^−4^ K^−1^. Although F depends on G, these parameters are largely controlled by chemical bond type (Figure 5). The trends in Figure 5 partly stem from the form of (21), because the size of F (or F*) depends on the size of G. The different trends in Figure 5 are not associated with the H*T* term, since each curve has some phases with H = 0 and some with finite H. The metals adhering to (23) occupy a different trend than insulators. We did not include metals with Curie transitions, as this magnetic change in *D* precludes a simple fit. Detailed discussion of insulators is given in [4].

#### 4.1.3. High Temperature Behavior

Whether the H*T* term is needed depends on the temperature reached, and also the size of G. The parameter H increases with G [4] (Figure 7.7b). This is a consequence of a power law with large G providing a flat trend at high *T*: hence, large H is required to observe the “bowing” of Figure 4 when G is large. Thus, for alkali halides, with high G-values, H must be large to be resolved, but for PbS with low G and high *T*_max_, H was essential to the fit. On this basis, the H*T* term may exist for ionic diatomic compounds, but is tiny, and melting occurs before sufficiently high *T* is reached.

#### 4.1.4. Vibrational vs. Electronic Transport in Metals

**Figure 6:**
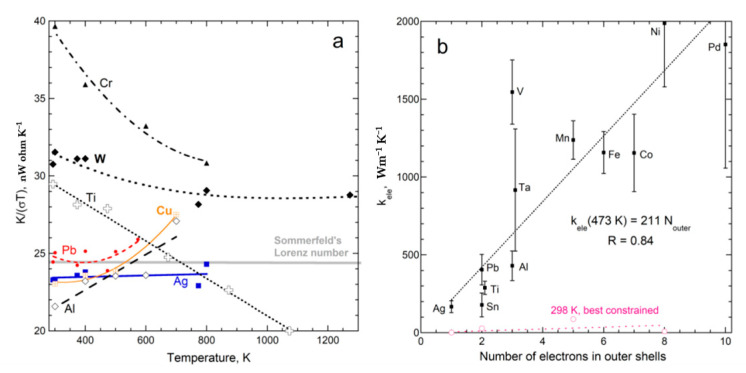
Data on metals: (**a**) Non-adherence to the Wiedemann–Franz law. Grey = Sommerfeld’s Lorenz number for the ratio of measured κ to electrical conductivity (σ) times T, is low by a factor of 3, stemming from ETKG describing fluctuations, not heat flow down a thermal gradient [6]. Black symbols and curves = data on 7 elements from [45] (Table 14.2). These elements had at least 4 temperatures where κ and σ were independently measured. (**b**) Correlation of κele (obtained from measured D and electronic heat capacity) with the number of nearly free electrons. Part (**b**) is modified after Figure 20b in Criss and Hofmeister [9], which is open access.

Metals have domains, even in nearly pure, elemental forms. Alloys are quite complex. Although much data exists on metals, *κ* is mostly measured and sometimes this is computed from electrical resistivity using the Weidemann–Franz law, despite its known inaccuracies [24], illustrated in Figure 6a. Additional data from [45] on Mg, Zn, Mo, Hf, Pt, Au, Ta, U show similar divergent trends [22] (Figure 9.10). Silver is apparently the only metal with a flat, linear fit.

Direct measurements of *D* vs. *T* for elements show various behaviors consistent with a vibrational mechanism (Figure 4; for details, see [9,22]). Importantly, (21) describes many metals, where G = 0, and so the H*T* term dominates. However, some metals show a linear decrease in *D* (e.g., copper [46]). Those with Curie points have complicated curves for *D*(*T*). Additional data are needed to delineate vibrational transport in metals and alloys, particularly as purification techniques have greatly improved since the 1970s when much data on bulk materials was collected. 

Regarding electrons, their rapid speeds limit our detection of this signal since our apparatus has a minimum acquisition time of 50 ms (Figure 1 and Figure 3). The signal could not be better resolved with longer samples, since very long lengths extinguish the flow of the electrons via interactions with the alternating current set up by vibrating cations. High temperature also squelches electronic transport because large numbers of thermally excited conduction electrons promote interference with each other. Despite the restrictions on electronic transport, consistent behaviors of *D*_ele_ and *κ*_ele_ were observed, as shown in Figure 6b, and many other figures in [9]. The results are summarized in [22].

### 4.2. Dependence of Thermal Diffusivity Near 298 K on Sample Thickness

Since thermal diffusivity and thermal conductivity have been viewed as material properties, few have considered that either of them depends on length. The exceptions are studies of thermal barrier coatings, which are defined by their thickness [47]. Here, we study much thicker samples than these films, while focusing on *L* below that typically used to study bulk materials. Fourier’s Equation (3) applies to our *T*-*t* curves (Figure 1 and Figure 3) because, in LFA, *L* is fixed and *T* varies little, so *D*, being constant during the experiment, is a reasonable approximation.

#### 4.2.1. Effect of Thickness on D of Insulators at Ambient Temperature

**Figure 7:**
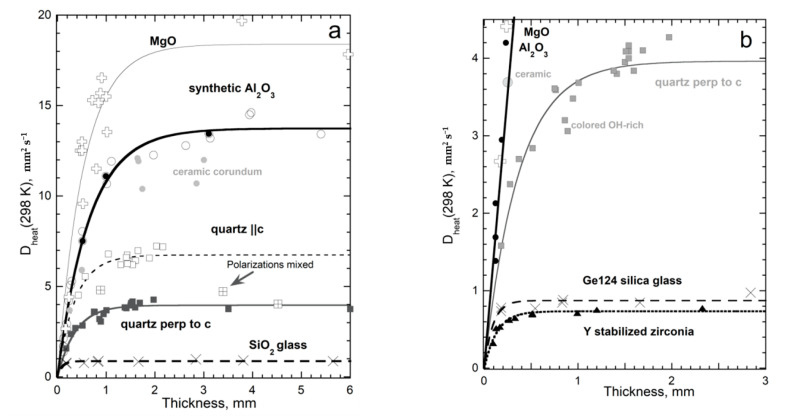
Dependence of *D*_heat_ at 298 K on thickness. Fits are least squares to (22): (**a**) High *D* samples. Not shown is a point for MgO at 17.5 mm^2^ s^−1^ for *L* = 10.0 mm. Open plus = MgO. Circles = corundum, with black dots representing (1120) orientations. Grey dots = ceramic Al_2_O_3_. Squares = two orientations of quartz. X = Ge124 silica glass. (**b**) Expanded view showing low *D* and emphasizing thin samples. Triangles = yttrium stabilized cubic zirconia. These samples are fairly hard, permitting the preparation of sections approaching *L* = 0.1 mm at 5–6 mm across. Soft materials (alkali halides and micas), shown previously [4], are omitted due to difficulties in preparing very thin sections with parallel faces. Modified after Figure 7.9 in Hofmeister [4], with permission from Elsevier.

Synthetic MgO and Al_2_O_3_ are focused on because impurities are negligible [10]. To avoid the effects of variable protonation on silica glass, only dry (35 ppm OH) Ge124, which is a fused quartz [44], was studied. Natural quartz has variable, low amounts of cation impurities, whereas synthetic quartz has significant amounts of hydroxyl [34], which both affect *D* and thus cause scatter in measured values (Figure 7).

A wider range of lengths than in the abovementioned original publications were examined by [4], and some measurements were added on yttrium-stabilized cubic zirconia (YSZ), a technologically important thermal barrier coating. These YSZ crystals have typical impurity contents of ~8 wt % yttrium oxide and ~1 wt % hafnium oxide. Here, we add measurements of very long and very short quartz (citrine and Hot Springs #2 [34]; *T*-*t* curves in Figure 1e) plus measurements of blue and green hydrothermal quartz with *L* ~ 0.9 mm, and data on ~6 mm long glass (Ge124).

Thicknesses over ~30% of the sample diameter enhance two-dimensional cooling to the surroundings, decreasing the accuracy of our measurements at large *L*. Nonetheless, an asymptote describes large *L* for all samples. The asymptote has little scatter for isotropic glass and YSZ (Figure 7), due to these having reasonably large aspect ratios. The odd point for MgO probably involved surface hydration, since the other samples were either measured shortly after opening sealed containers or were thinned by grinding. The cause of scatter for synthetic sapphire is unclear; however, they may be connected with aspect ratios or with impurities, as Cr is common, but was not measured. Scatter is not caused by orientation, since heat flow perpendicular and parallel to the c-axis are similar. A few large sapphires had birefringence due to strain. Note that the thick samples of quartz-oriented ||**c**, which is the fast direction, merged with *D* values for samples oriented ||**a**. This behavior is consistent with polarization mixing, seen in various studies of natural olivine, (Mg_0.9_Fe_0.1_)_2_SiO_4_, which involved measuring samples of various mm lengths and aspect ratios (tabulated in [33]).

A single, simple formula describes *D* for all insulators studied, when a wide range of thicknesses was used and impurity content varied little:

(22) $$D(L,298K)=D_{\infty }\left[1-\exp(-bL)\right]$$

The parameter *D*_∞_ (Table 2) represents thermal diffusivity at 298 K for a very long sample whereas parameter *b* represents attenuation of the heat transmitted. The thickness separating “thin” from “thick” behavior increases with *D*_∞_ for *T* = 298 K and is reflected in the inverse correlation of the parameters for the crystals (see below). Attenuation for silica glass is not well constrained, as the “thin” regime was barely reached.

Equation (22) provides *D* = 0 at *L* = 0, which agrees with the limits of (7), obtained in dimensional analysis, and shows that heat cannot be diffused if no medium exists. These data demonstrate that neither *D* nor *κ* are material properties and indicate that the microscopic mechanism for conduction is diffusion of light.

#### 4.2.2. Effect of Thickness on D of Elements and Alloys at Ambient Temperature

**Figure 8:**
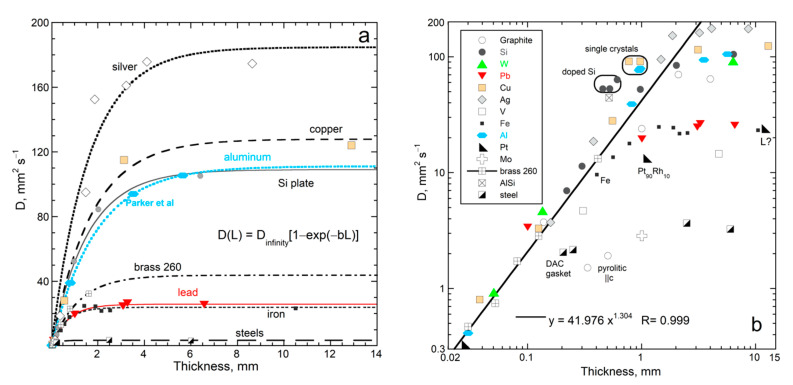
Dependence of *D* at 298 K on thickness for various elements and alloys: (**a**) Linear plot, showing fits to (22) for elements with >4 measurements over a wide range of *L*. Parker et al. [11] used an adiabatic model, rather than Cowan’s model used here. (**b**) Expanded view on logarithmic scales showing additional materials, mostly foils, and some single crystals. The power law fit is to brass and only for *L* < 0.5 mm. The steels are non-magnetic. Foils were obtained from Alfa/Aesar, the Washington U. Physics Department machine shop, and various other sources.

The thermal diffusivity of Si, graphite, nearly pure metals, and alloys (semiconductor data from [4,9]) depend on *L* at 298 K (Figure 8a) in a manner similar to the insulators. Likewise, these data are well-described by (22) and the coefficients are inversely correlated (Table 2). For raw *T*-*t* curves, see Figure 1a. Graphite, although nearly pure, was not fit due to variable porosity and directional heat flow, which greatly affects the pyrolytic chips (Figure 8b). Single crystals of Cu and Al lie above the trend for their grainy counterparts, consistent with grain boundaries scattering light. Regarding Si, grinding one slab provided a different trend than that of variously doped samples, which should have different near-IR spectra. Very thin Si has ballistic radiative transfer in the near-IR at 298 K, but this contribution was excluded from measured *D*-values.

Ductile metals and alloys are widely available as foils. All very thin elements and alloys roughly follow the power law defined by brass alloy 260 (Figure 8b). Because the results for sub mm thicknesses do not depend strongly on chemical composition, we did not perform chemical analysis.

**Figure 9:**
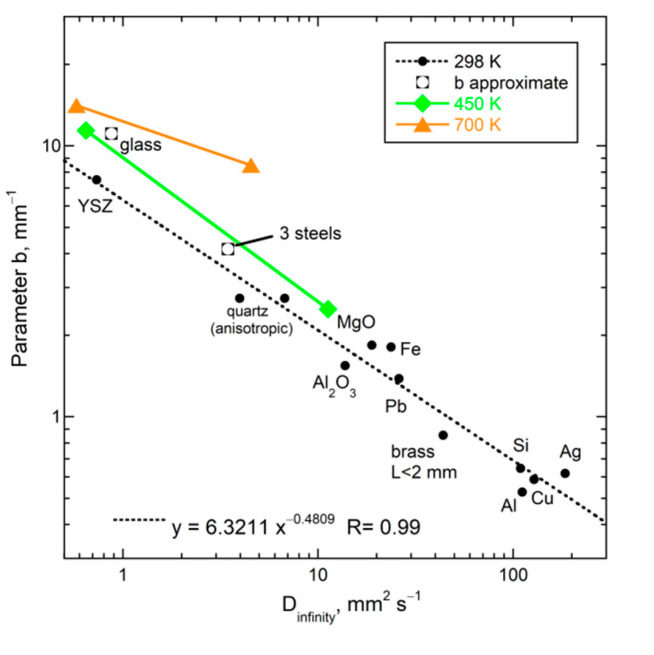
Logarithmic plot of fitting parameters in Table 2. Fused quartz and steel were not used in fitting because these measurements did not include very thin samples. High *T* data are shown only for MgO and YSZ.

The fitting parameters of Equation (22) in Table 2 are correlated (Figure 9). The same power law holds for insulating oxide crystals, semiconducting Si, and metals. Thus, the same mechanism operates for these substances with very different chemical bonding. Criss and Hofmeister [9,22] present diverse evidence for heat being transported by vibrations in metals, except for a very short time following the laser pulse, where rapid electronic transport occurs under disequilibrium conditions—Figure 3 and Figure 6 show some of the evidence. Pressure studies provide further confirmation (Section 5 and Section 6).

### 4.3. Combined Effect of Thickness and Temperature on Thermal Diffusivity

Observation of nearly constant *D* near 298 K for thicknesses exceeding ~1 mm (Figure 7 and Figure 8) explains similar, but not exactly equal, results on the same material from different laboratories (presuming removal of ballistic transport). Thickness is infrequently reported (e.g., [49]), but is usually 1 to 5 mm. Therefore, this section focuses on *D*(*T*,*L*) for samples that are thin, but still represent bulk material. The results are presented only for electrical insulators, because these uniformly follow Equation (21) for *D*(*T*) at large *L* (Table 1; Figure 4). Thick metals and alloys show a wider range of high-*T* behaviors, which requires studying many materials, and is beyond the scope of this report.

Several samples studied previously, if thin, differ from the trend in Figure 4 by having *D* that initial increases with *T* above ~290 K, but only over a small *T* range, and then decreases. Additional examples of upturns exist where only a few thicknesses were studied: these are polycrystalline diamond [10] (Figure 2f); Si [4] (Table 7.2); SiC [4] (Figure 7.3); muscovite-F within the layers [4,50].

**Figure 10:**
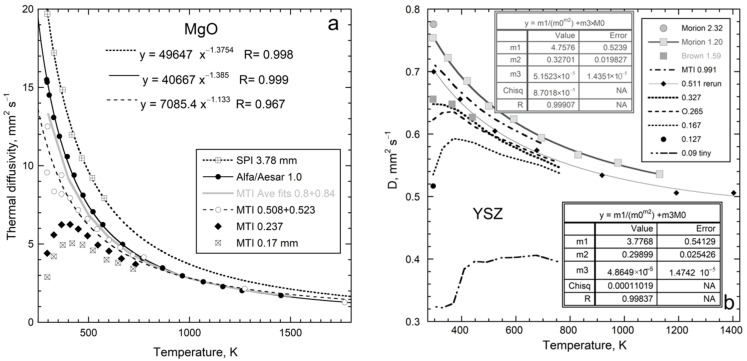
Thermal diffusivity of cubic insulators vs. temperature for various thicknesses. Insets list samples from different sources and their thicknesses, plus fits: (**a**) MgO, where surface hydration is possible. Some datasets with similar thickness were merged, as indicated in the inset. The ~0.5 mm samples diverge from the power law but are still reasonably fit. (**b**) Yt-stabilized cubic zirconia. Colorless samples with *L* < 1 mm are from MTI Corp., whereas thick samples are from Morion Company. Lower *D* for brown colored YSZ from Pretty Rock Inc. is attributed to additional impurities. Solid lines are least squares fits to the high-*T* datasets using Equation (21). Data on the thinnest section is more uncertain, due to lateral size. However, even with an uncertainty of 20%, these *D*-values remain below trends for larger samples.

Results for MgO and cubic zirconia (Figure 10) are consistent with detailed measurements of *D*(*T*) for various lengths of synthetic sapphire [4] (Figure 7.10a), which showed that the upturn shifts to higher temperature as *L* decreases from ~½ mm, making the peak more pronounced. This behavior explains why sapphire with *L* near 1 mm did not follow the universal form Equation (21), unless its three lowest *T* data points were omitted from fitting, as done in Figure 4. Similarly, Equation (21) applies to sections of sapphire with *L* = 0.5 mm if its six lowest *T* data points are omitted when fitting.

We have not amended empirical Equation (21) to portray the upturns. Establishing a complicated empirical formula requires cryogenic measurements on a variety of substances. Roughly, *D*(*T*) at low *T* for thin samples points toward the origin. This linear increase in *D* with *T* indicates radiative transfer at low *T* in thin samples, for the same reasons that the H*T* term indicates radiative diffusion at high *T* in thick samples [4].

**Figure 11:**
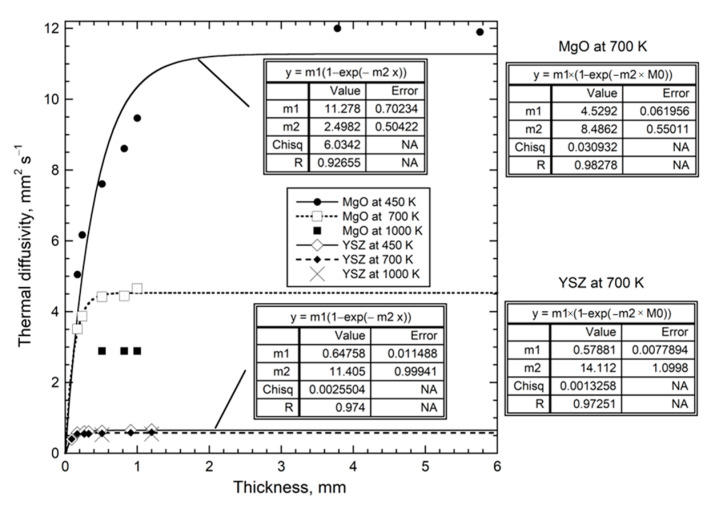
Thermal diffusivity of MgO and YSZ as a function of thicknesses at various temperatures. Insets list fits. Samples are described in Figure 10 and previously in [4,10].

High temperature results for *D*(*L*) in Figure 11 are fit with Equation (22). The trends defined by MgO and YSZ at 450 and 700 K are both parallel to results at 298 K (Figure 9). Although more data are needed, the plots in this section show that the dependence of *D* on *L* and *T* is consistent, where the phase controls the long length asymptote of *D*_∞_, which then controls the attenuation parameter. This control stems from the limits of Equation (7) and the forms of Equations (5) and (15).

## 5. Heat Conduction at Elevated Pressure

In high pressure measurements of heat transport, physical contacts are unavoidable, and cause thermal interface losses, as is long known. Compression, improving contacts and reducing pore space in grainy materials, is detrimental as this alters pressure derivatives from those intrinsic to the material. Regarding insulators, spurious radiation increases as *L* decreases. Hence, experimental uncertainties are at least as large as those associated with the method and material near 1 atm of, typically, 5 to 20%. Commonly applied methods have been covered in detail [17,19]; the results are compared in [4] (Chapter 7). However, earlier assessments did not consider the consequences of length-scale dependent physics.

Fortunately, measurements up to 2 GPa are negligibly affected by *D* and *κ* depending on *L*, since ~mm scales are probed. In contrast, high pressure studies using ultrathin samples in diamond anvil cells (DAC) are greatly impacted. This has not been recognized due to the common practice of benchmarking against data on thick samples. Additionally, ballistic contributions are enhanced for thin insulators. Below, we separately evaluate datasets with long and short length-scales, and then infer lifetimes and mechanisms from reliable values of ∂ln(*κ*)/∂*P* from 26 different thick solids. For brevity, alloys are not distinguished from metals in this report, despite their complexities.

### 5.1. Low-Pressure Transport Data on Thick Metals

Either the hot wire/hot strip or Angstrom’s technique constrains thermal transport in metals at low *P* (<2 GPa) and low *T* (<1000 K) since metal–metal contact losses are low, ballistic radiative transfer gains are negligible, and length scales are large, as in 1 atm studies. Table 3 summarizes the results at 298 K. Bridgman’s [51] pioneering study on many different samples of Pb and Sn is included, since averaging reduces uncertainties.

### 5.2. Low Pressure Transport Data on Thick Electrical Insulators and Si

Table 3 summarizes reliable data on mm length-scales obtained near 298 K in piston-cylinder or multi-anvil devices using the hot-strip/hot wire or Angstroms’ methods. Samples are single crystals, glasses, with some measurements of disks of fine-grained powder that were compacted prior to study. Unlike metals, systematic errors exist due to interface thermal resistance and ballistic transfer. Since optical methods minimize these two systematic errors, such studies are included for comparison, despite experimental uncertainties existing from the use of thin samples. Although multi-anvil studies using Angstrom’s method do not account for the pressure medium, relative values should be less affected than absolute, permitting the extraction of ∂ln*κ*/∂*P*. These studies [52,53,54] are included when data were collected sufficiently close to ambient conditions to warrant linear extrapolation to the limit *P* = 0. Agreement among studies is quite reasonable (Table 3).

To evaluate pressure effects on *κ* from the most accurate data in existence, Table 3 excludes studies that use approximate formula for the hot wire approach. Comparative methods are omitted because quartz, which has large ballistic effects, serves as the standard. Table 3 excludes early studies with less reliable pressure determinations, and studies that exhibited hysteresis (e.g., NaClO_3_). Additionally, also excluded are coarse-grained samples due to their high porosity, and materials with a phase transition close to 298 K (e.g., sulfur). Data on soft alkali halides with *B_T_* < 14 GPa [76,77] are omitted because deformation around the embedded wire during compression is possible. Soft alkali halides are hydroscopic, which could influence their heat transport. Also, transformation to the B2 structure at low *P* limits accuracy for the derivative. Hydroscopic RbF is included, because this is relatively hard, but its thermodynamic properties are uncertain [68].

### 5.3. Problematic DAC Studies of Very Thin Metals

**Figure 12:**
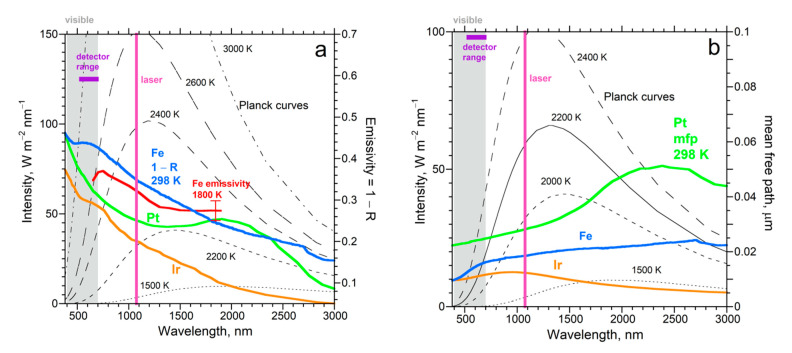
Spectra in the visible (grey rectangle) and near-IR (white background) wavelength ranges relevant to laser heating of foils. Thin patterned black curves = blackbody emissions with *T* as labeled. Green, blue or orange curves = metal spectral properties calculated from indices of refraction tabulated in [83]. Pink vertical line = the laser wavelength. Purple horizontal bar = range of the detector used in [30,81,82]: (**a**) Comparison with high-*T* emission data (red curve) from [84] to 1-*r*, which is reasonable for metals, due to opacity; (**b**) Comparison of mean free paths (=1/*A*) to blackbody curves for temperatures commonly explored in laser-heating DAC studies.

For *L* ~ 4 μm used in diamond anvil cell studies, *D* near 0.03 mm^2^ s^−1^ is expected at ambient conditions for any metal (Figure 8). Values of *κ* that are 100-fold larger, as in conventional studies of large samples, are reported for Fe and Pt foils compressed to ~130 GPa [81,82], whereas rates in Table 3 suggest only a 5-fold increase. To understand this discrepancy, spectra (Figure 12) are used to interpret thermal evolution that is implicit in the author’s raw data (e.g., Figure 2b).

#### 5.3.1. DAC Experiments with One Laser

In single laser DAC studies, *T* is obtained by fitting emissions over a narrow frequency range from an *L* = 0.1 μm uncoated iridium foil imbedded in an electrical insulator (e.g., MgO, an alkali halide, or silicate with the perovskite structure [30,85,86]). Extreme variations in *T* over short intervals (Figure 2b) and penetration of the laser directly into the bare foil prevent accurate determination of *D* or *κ*. However, information can be gleaned on the propagation of laser light through the foil.

Calculated temperatures vs. time resemble Cowan’s model (Figure 2a) because both situations entail strong ballistic radiative cooling of the sample to the surroundings, which moderates any possible heating. The rear foil surface warms nearly simultaneously with the applied pulse (Figure 2b). This surprising response is explained by the schematics of Figure 2c,d and spectral data. Because the foil is not graphite-coated, laser light (1064 nm) penetrates the foil. Attenuation of the signal by 1/e occurs over a distance of 1/*A*, which is 0.012 μm for Ir at ambient conditions (Figure 12b), and should be similar at high *T* and *P*. Rear side emissions likewise originate within 1/*A*. As laser light crossing the foil is attenuated, it is converted to heat (Figure 2d). However, any heat produced is outpaced by the travel of the laser beam because time is needed to absorb light with a single frequency and then to convert this energy to thermal emissions. Figure 1 and Figure 3 demonstrate that this conversion takes time. Thus, the immediate heating of the rear surface in these single-laser experiments [30,85,86] results from the crossing of laser light. Although its attenuation is strong, ~1/e^8^, laser light is intense. Diffusion of heat also occurs, which later arrival is masked by ballistic cooling to the surroundings (Figure 2b).

To estimate *D*, we apply Cowan’s model to the cooling part of the curve. For strong radiative cooling, 5*t*_½_ occurs near ½*T*_max_ (Figure 2a). Figure 2b suggests that 5*t*_½_ = ~50 ns, and so *t*_½_ = ~10 ns. For strong radiative losses, the numerical factor of 0.139, pertaining to adiabatic transport in (15) should be replaced by 0.08 [29] (Figure 3), yielding *D* = 0.08 mm^2^ s^−1^. Albeit rough, our estimate for *D* of Ir at high *P* and *T* is compatible with data in Figure 8 and Table 3, and minor changes in *D* with *T* (Figure 4).

#### 5.3.2. DAC Experiments with Two Lasers

**Figure 13:**
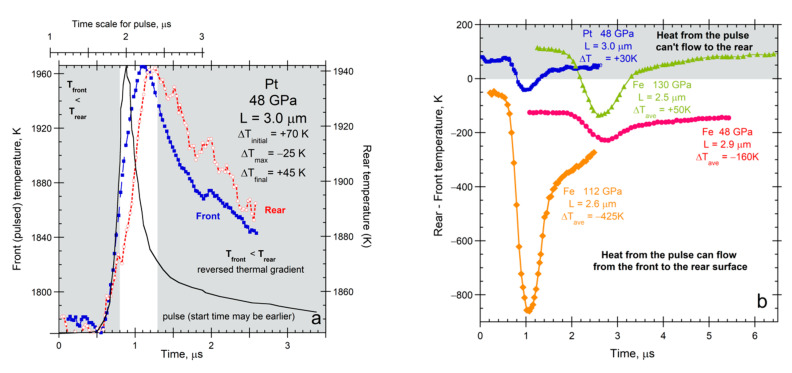
Temperatures calculated in two-laser DAC experiments: (**a**) Example from [81], digitized and rescaled. Squares represent the front surface which absorbs the laser pulse and circles represent the rear. Conditions and *T* differences are listed. Y-axes limits were chosen so each *T*-*t* profile fills the graph. Platinum experiments [81] began with the rear surface being hotter (grey rectangle), which prohibits the diffusion of heat from the front to the rear but permits the travel of the laser beam. Thin black curve = laser pulse profile [81]. Since the relationship of data collection to the pulse was not specified, the laser profile was placed where the front surface began warming. Part (**a**) was modified after Figure 3d,f of McWilliams et al. [81], with permissions from Elsevier. (**b**) Temperature differences across the ~3 μm foil of Pt compared to those in experiments on iron [82], obtained from digitizing and subtracting temperature curves in [81,82]. Grey rectangle shows times where the rear surface is undesirably hotter than the front. Average *T* differences are rough.

References [81,82] ascertained temperatures as a function of time by fitting emissions in the visible region from each side of uncoated Fe and Pt foils that were embedded in a medium at high *P,* as sketched in Figure 2c. Thickness is initially 4 μm from their text and tables, so Figure 1 of [82] has incorrect dimension(s). A continuous wave laser is split to irradiate both sides of the foil, which establishes an initial temperature difference of +110 to −125 K across *L* (Figure 13). Subsequently, the front foil surface was irradiated with a ~½ μs wide pulse from a second infrared laser. None of the requirements for LFA (the bullet list in Section 3.1) are met.

Published temperature calculations are scaled (Figure 13a) to emphasize delays in heating the rear side relative to the irradiated front side. Initially, Pt at 48 GPa [81] had a front surface that is undesirably cooler than the rear (Figure 13a). Net heat cannot be conducted from the front to the back, per Fourier (2), yet the emissions of both surfaces initially rise together. Conduction is impossible over ~30% of the time that the rear surface temperature climbs. Therefore, emissions from the rear surface increase, not from conduction, but rather from direct receipt of the laser pulse.

As in the single-laser experiments (Section 5.3.1), the rear surface is initially warmed by laser light penetrating and crossing the uncoated foil. Although the pulse creates heat as it progressively attenuates, this heating front arrives later. Once *T* of the front surface exceeds *T* of the rear, heat can diffuse between these two surfaces. However, conditions permitting diffusion from front to back surfaces exist only for ~½ μs in the experiments of [81], during which time period the pulse subsides and the foil radiatively cools. Radiative cooling is guaranteed by the transparency of the pressure media. Note that a metal foil was used in the one-laser experiments [30,85,86] because their MgO and alkali halide samples would not otherwise have been heated by the laser.

As Cowen demonstrated, when surface radiative cooling is strong, it occurs over the entire experiment (Figure 2a). That heating of the rear foil surface scarcely lags heating of the front (Figure 13a) confirms that the key heating mechanism of the rear surface is by the impingement of attenuated laser light. The rather constant lag time, while both surfaces warm, is consistent with conversion of laser light to thermal energy in the solid requiring some finite time, as demonstrated in LFA (Figure 1 and Figure 3).

Similar experiments at 130 GPa on Fe [82] likewise have an initial thermal gradient that prohibits heat conduction from front to rear (Figure 13b). Later, rearward heat conduction is permitted over a ~1 μs time period. The time response of Fe has a longer lag time than for Pt (not shown), which is consistent with the stronger attenuation of laser light in Fe than in Pt (Figure 12b). The shorter mean free path in Fe causes a more gradual *T* increase in the rear surface with time, supporting that the heating of the rear surface is a direct response to the laser beam. For Fe at 48 GPa, the rear surface always has lower *T* than the front (Figure 13b). Despite rearward conduction being permitted at all times, the *T* rise for the rear surface differs little from the rise under a “reversed” thermal gradient (cf. red and blue curves in Figure 13b). At 112 GPa, a favorable *T* gradient exists (orange curve). Although the gradient prior to the pulse was smaller by a factor of two compared to the 48 GPa run, whereas the increase in *T* for the front face was larger by a factor of 4, the thermal response of the rear face at 112 GPa is similarly shaped to the response of the rear at 48 GPa and the *T* increase was not much more (see graphs in [82]). Clearly, ballistic radiative cooling dominates these experiments.

In summary, similar shapes occur for rear surface heating curves whether or not conduction of heat from the pulse is possible, whereas rear surface cooling curves have slightly different shapes, which depend on which side is hotter from continuous heating (Figure 13b). These observations require that the attenuation of laser energy (a spike near ~1000 nm) as it crosses the sample is the mechanism that primarily heats the rear surface, rather than the diffusion of heat.

Neither of the references [81,82] mention the sign variations of the temperature gradient maintained by the continuous wave laser, which sometimes prohibits conduction rearwards. The authors unjustifiably claim that their experiments are analogous to LFA. The authors view radiative cooling as being absent from their experiments, which contradicts the seminal work of Cowan, spectral characteristics of the media, and assumptions they made in modeling emissions. Reference [81] mathematically “broadened” the laser pulse to better match the rise in *T* for the front surface (Figure 13a). The shape difference corroborates that time is needed to convert laser energy to heat energy inside the front face of the foil, while the parallel shapes for the front and rear supports that the rear surface is directly heating by laser light.

Based on incorrect assumptions, a single value of *κ* is extracted from each DAC run [81,82]. Although a single value of *D* can be extracted from LFA, this is due to nearly isothermal conditions and accounting for radiative losses in thermal models of the *T*-*t* curves (Section 3; Figure 1 and Figure 3), where Cowan’s ℘ parameter (Figure 2a) is utilized, albeit not reported. Hence, modeling heat transfer in these DAC experiments requires, as a minimum, accounting for the temperature and time dependence of radiative losses, addressing ∂*κ*/∂*T* for segments involving conduction, accounting for the direction of heat flow, and addressing attenuated laser light heating the rear side.

### 5.4. DAC Studies of Thin Electrical Insulators

Single-laser heating experiments probe processes inside the embedded foil and ballistic cooling, not conduction into the surrounding insulator, as claimed in [30,85,86]—see [87]. This finding is based on use of containers in LFA. When the absorbing surface is the sample front (not the container front), the conductive properties of the container are not germane.

Thermoreflectance has become recently popular for DAC studies. Such experiments involve thin layers, where the *C* and *κ* of these layers are required, as well as knowing thermal effusivity (Ε = *Cκ*) of the medium, thermal resistance of the interfaces, and the thickness of the metal coating [17]. The penetration depth of the heat into the sample is key to quantifying conduction, per Fourier, but this depth is not measurable. Multiple pulses are applied sequentially and averaging is used. Analyses are neither simple, nor related to a measurable length scale of the material, but instead are based on either the time or frequency response to the pulses. A variation used in DAC experiments, where the probing lasers are applied to opposite sides of the sample [88,89], presents some problems [4] (pp. 125–128). The present paper focuses instead on sample size, which has not been previously discussed. The effect of length-scale physics is gleaned by examining a DAC study using a standard approach.

Hsieh et al. [90] studied flow along the c-axis of muscovite crystals with *L* = 20 μm. They obtain 0.47 Wm^−1^ K^−1^ at ambient conditions, which matches 1977 conventional measurements [91]. This value is appropriate for larger *L*, near 200 μm [4,50]. Allowing for slight variations in chemical composition indicates that *κ* from [90] should instead be near 0.05 Wm^−1^ K^−1^.

To evaluate their high *P* acquisitions, Hsieh et al. [90] included the response of heat capacity to pressure of Ar, Al, and muscovite, which were considered to positively increase by ~10% over 10 GPa. Instead, the thermodynamic identity:

(23) $$\frac{\partial c_{P}}{\partial P}=-TV\left(\alpha^{2}+\frac{\partial \alpha }{\partial T}\right)\;\mathrm{or}\;\frac{\partial \ln(c_{P})}{\partial P}=-\frac{\gamma_{th}T}{B_{S}}\left(\alpha +\frac{\partial \ln(\alpha )}{\partial T}\right)$$

provides a negative sign (Table 3). Positive signs link to bond bending (Section 5.5) that is not present. Instead, micas readily compact parallel to the c-axis by contraction of weak van der Waals bonds that link the structural layers. Using an incorrect sign for ∂ln*c_P_*/∂*P* leads to incorrectly positive ∂^2^*κ*/∂*P*^2^ and excessively large ∂*κ*/∂*P*. However, correcting *κ*(*P*) is precluded because erroneous signs for ∂ln*c_P_*/∂*P* of Al and Ar were also used in [90]. Lastly, the Al coating is very thin, but thermal modelling in thermoreflectivity studies rely on large *κ* for Al that pertains only to thick samples.

The many parameters used in data analysis in thermoreflectance studies, including estimates for unknown distance over which diffusion occurs, underlie reported *κ*-values that agree with previous studies of bulk material. All thermoreflectance studies involve thin layers and should instead provide very small *κ* and *D* (Figure 7, Figure 8, Figure 9 and Figure 10). Due to heat transport depending on length, coupled with modelling problems, values of *κ* and its pressure derivatives reported in thermoreflectance experiments using thin samples (e.g., [90,92]) are problematic.

### 5.5. Implications of Reliable Data on ∂ln(κ)/∂P for Lifetimes and Mechanism

Table 3 compiles accurate data on 26 different samples, where a few compositions were measured twice. This database covers metals, alloys, Si, CaF_2_, alkali halides in B1 and B2 structures, simple and complex oxides, plus fused quartz. Their thermodynamic properties are well-studied.

#### 5.5.1. Mechanism Independent Information on Lifetimes and Compression

The definition of *κ* for an isotropic material and dimensional analysis (*D* ∝ *L*^2^/τ, (5)) yield:

(24) $$\frac{\partial \ln\left(\kappa \right)}{\partial P}=\frac{\partial \ln\left(\rho \right)}{\partial P}+\frac{\partial \ln\left(c_{P}\right)}{\partial P}+\frac{\partial \ln\left(D\right)}{\partial P}=\frac{1}{3B_{T}}+\frac{\partial \ln\left(c_{P}\right)}{\partial P}-\frac{\partial \ln\left(\tau \right)}{\partial P}$$

where *B* is the bulk modulus. Using logarithms removes constants, while reducing the effect of small experimental uncertainties. Unlike previous efforts, we did not use dimensionless relationships for *D* that incorporate carrier speed or mean free path (mfp). Using speed × mfp depicts the long times between collisions, rather than the short time of the interaction during which heat is absorbed. The shorter lifetime dominates since lifetimes are the inverse of probabilities [8]. Utilizing *D* ∝ *L*^2^/τ addresses length-scale physics of heat transport processes (Section 3 and Section 4).

For crystalline materials, the compression term is small and positive (~0.3% GPa^−1^), whereas the heat capacity term, either from (23) or measurements, is similarly small (Table 3). Lifetimes calculated by difference from (24) decrease with pressure (Table 3, right-most column). Negative ∂*τ*/∂*P* results because compression concentrates matter inside the solid and, therefore, increases the probability of its interaction with heat flowing through.

Silica glass contracts by bond bending, providing negative thermal expansivity below ~220 K, but a positive ∂α/∂*T* derivative near 298 K (summarized in [44]). Equation (23) gives ∂ln*c_P_*/∂*P* = −0.028% GPa^−1^, which differs slightly from experimental determinations of [66]. For crystals, (24) gives positive ∂ln*τ*/∂*P*, ranging from +1.9 to 5% GPa^−1^ (Table 3). For fused quartz, pressure compacts the volume at the expense of lengthening primary bonds, whose vibrational transitions uptake and release heat. The weakening of bonds (and thus interactions) during compression causes lifetimes to increase with *P*.

**Figure 14:**
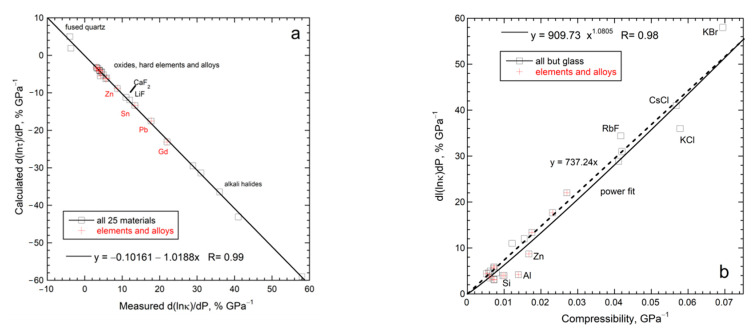
Graphical representation of data compiled in Table 3. Metals and Si (red crosses) are included in the fits: (**a**) Comparison of values from Equation (24) to measurements of *κ* as a function of pressure. RbF was excluded due to uncertainites in *c_P_* [68]; (**b**) Dependence of ∂ln(*κ*)/∂*P* on compressibility (*B_T_*^−1^), excluding fused quartz where bonds bend rather than contract. In both panels, hard solids cluster. These involve small, difficult to measure, changes during compression. Soft halides have large changes, but deform and absorb water, producing systematic errors. Halides with *B_T_* < 15.5 GPa, which is accompanied by ∂ln*κ*/∂*P* > 60% GPa^−1^ and phase transformations at very low *P*, were excluded from these plots.

Figure 14a shows that all 26 solids of Table 3 follow:

(25) $$\frac{\partial \ln\left(\tau \right)}{\partial P}=-\frac{\partial \ln\left(\kappa \right)}{\partial P}$$

Thus, thermal conductivity embodies how the solid interacts with any heat flowing through. The negative sign results from lifetimes being the inverse of probabilities. More can be gleaned:

#### 5.5.2. A New Thermodynamic Formula

The trend of Figure 14a for large samples is possible if and only if the following relationship holds:

(26) $$\frac{\partial \ln\left(c_{P}\right)}{\partial P}=-\frac{1}{3B_{T}}=\frac{\partial \ln\left(L\right)}{\partial P}=-\beta_{L}$$

where *β_L_* is linear compressibility. Existing thermodynamic identities [93] do not include Equation (26). Isothermal conditions apply, which seems counterintuitive, but constant *T* describes situations where heat being applied matches heat lost to the surroundings via ubiquitous blackbody emissions.

Heat flows down the temperature gradient, constituting a 1-dimensional process. Thus, Equation (26) states that, during heat flow at some given *T* while varying *P*, the solid loses heat because the mass is compacted while the volume of the light is unchanged. Only one direction pertains because heat flow is one-dimensional. Regarding uniaxial tin, the experiments were on polycrystalline metal [51], and so Equation (26) also depicts directionally averaged material. For anisotropic material, the direction should matter—this can be experimentally tested.

Additional tests of Equation (26) are required to ascertain generality and need not involve heat transport measurements. However, this is beyond the scope of the present report.

#### 5.5.3. Constraints on Bridgman’s Parameter

Bridgman’s parameter is defined as:

(27) $$g=\frac{\partial \ln\left(\kappa \right)}{\partial \ln(V)}=-B_{T}\frac{\partial \ln\left(\kappa \right)}{\partial P}$$

The linear fit in Figure 14b (which involves percentage changes) indicates that Bridgman’s parameter equals ~7.3 for diverse solids. Consequently:

(28) ∂ln(τ)∂P=gBT≅7+13BT=~7BT+βL

Equation (28) suggests that a universal mechanism for heat transfer exists for solids when *L* is sufficiently large that heat transfer is diffusive. Section 6.2 relates *g* to thermodynamic properties in addition to *β_L_*.

#### 5.5.4. Mechanisms of Heat Transport

The Weidemann–Franz law represents an electronic mechanism in metals:

(29) $$\kappa_{\mathrm{ele}}=\frac{\pi^{2}}{3}\frac{k_{B}^{2}}{e^{2}}\sigma T$$

where *k*_B_ is Boltzmann’s constant, *e* is electron charge, and *σ* is electrical conductivity. A logarithmic derivative is unaffected by geometrical factors or physical constants:

(30) $$\frac{\partial \ln\left(\kappa_{ele}\right)}{\partial P}=\frac{\partial \ln\left(\sigma \right)}{\partial P}$$

Experiments show that the electrical conductivity of Gd and Pb decreases as *P* increases [94], as also occurs for AuCu_3_ and ordered AuCu [62]. However, *κ* increases with *P* for metals (Table 3). Even when both derivatives are positive, the 1:1 correlation of (30) is not followed: examples are Ni, Fe, Cu, and Zn (see references in Table 3). Nor does *κ* depend linearly on *T*, as in (29), the model failure of which is well-known (e.g., Figure 6).

Electrical resistivity (1/*σ*) describes impediment of flowing conduction electrons via their interactions with the valence electrons which oscillate with the nuclei, not the promotion of heat flow. Unlike charge, heat readily leaves a material. This important difference, perhaps prompted Maxwell’s remark that the analogy of heat flow with currents should be used with caution.

Heat enters and departs metals as light and is stored in their lattice vibrations. Compression affects heat storage and heat transport in metals, just as in insulators and semi-conductors (Figure 14). The thermal diffusivity and *κ* of metals depend on length-scale just as insulators and semiconductors do (Figure 7, Figure 8 and Figure 9). However, models based on phonon scattering preclude length-scale physics (see also Section 1). Foremost, in a gas, motions of heat and mass are indistinguishable, and thus a gas is not a suitable analog for the behavior of solids, where these two motions are independent. Unlike gases, heat transport in solids is disconnected with mass diffusion.

Based on heat and light being the same phenomenon in dilute media, as established via spectroscopy [95], heat diffusion in a solid thus stems from sequential absorption and the re-emission of IR light down the temperature gradient. This mechanism addresses the similar dependence of thermal transport properties of metals, insulators, and semiconductors on *T*, *P*, and *L* (above and Section 4). The departure of internal blackbody radiation from a heated solid underlies spectroscopic emissions measurements: this was used by Bates et al. [96] to derive exact formulae describing emissions from solids with varying thickness. Further discussion of radiative diffusion requires a quantitative model.

## 6. Radiative Diffusion Model for Conductive Heat Transfer in Solids

Formulae for the effective thermal conductivity of diffusing blackbody radiation are commonly applied to high temperature situations by considering absorption in the visible region. Most formulae concern the emanation of light from some point in space, rather than parallel rays in a Cartesian system, which describes most experiments. Although the mechanism of heat uptake is microscopic, the model is macroscopic since the absorption coefficient is a material property.

### 6.1. Basic Equations

Based on engineering and astrophysics textbooks [97,98], the diffusion of blackbody radiation per steradian in spherical geometry is described by:

(31) $$\kappa_{rad}(T)=\int_{0}^{\infty }\frac{1}{A(\nu ,T)}\frac{\partial I(\nu ,T)}{\partial T}d\nu =\int_{0}^{\infty }\frac{1}{4A(\nu ,T)}\frac{\partial I_{BB}(\nu ,T)}{\partial T}d\nu$$

Equation (31) also holds for one Cartesian direction [4] (Chapter 11). This idealization requires that the material is optically thick at all frequencies, i.e., light is attenuated through interaction with many, many atoms. Thermal resistance at interfaces is not addressed. Thus, Equation (31) requires large *L* at every frequency, is appropriate for single-crystals or glassy samples, and represents a single mechanism.

Incorporation of the index of refraction in Equation (31), as in the geophysics literature, is inappropriate for parallel rays. Multiplication by 4π describes total emanations of light from a point, not parallel rays. Division by three, as in some presentations, represents directional averaging during thermal fluctuations, as in EKTG, but is inappropriate for heat transfer down the thermal gradient.

The factor of 4 in the RHS arises as follows: Total flux (ℑ) from all frequencies from a point is:

(32) $$ℑ{\;(\mathrm{in}\;\mathrm{Wm}}^{-2}{\mathrm{sr}}^{-1})=\int_{0}^{\infty }Id\nu$$

Planck’s historic function is:

(33) IBB(ν,T)=2hν3c21exp(hνkBT)−1

From Marr and Wilkin [99]:

(34) $$\int_{0}^{\infty }I_{BB}d\nu =\frac{2\pi^{4}}{15}\frac{k_{B}{}^{4}}{h^{3}c^{2}}T^{4}$$

Total radiant energy per area per time:

(35) $$ℜ=\sigma_{SB}T^{4}=4\pi ℑ$$

where (*σ_SB_*) is an experimental constant, that was first ascertained by Stefan circa 1879. The modern experimental value of 5.670 × 10^−8^ Wm^−2^ K^−4^ is identical to:

(36) $$\sigma_{SB}=\frac{2\pi^{5}k_{B}^{4}}{15c^{2}h^{3}}$$

Comparing Equations (32)–(36) shows that Planck’s formula should be divided by 4. For Planck to have benchmarked his prefactor against Stefan’s result required a difficult integration, the achievement of which by Fikhtengol’ts [100] postdates Planck. Hence, a historic numerical error exists. Recent literature either overlooks the above discrepancy, or mistakenly equates ℑ with ℜ. An alternate derivation of the intensity distribution function [101], based on spectroscopy and thermodynamics, following the approaches of Wein and Weber, yields *I*_BB_/4.

Measurements of emissions have not constrained absolute values of *I*(ν,*T*) because ascertaining the frequency distribution requires a prism or beamsplitter, which refracts and absorbs, and thus spectroscopic experiments are more uncertain than total emissions experiments. Both measurements require estimating emissivity of the source at high *T* where its frequency dependence (Figure 12) is additionally needed to determine *I*. However, high *T* spectroscopy studies are rare (see [83]). Hence, the experimental focus has been on the shape of the curve for *I* [101].

#### 6.1.1. Geometrical Factors

The temperature derivative of *I*_BB_ is proportional to Einstein’s heat capacity:

(37) $$\frac{\partial I_{BB}\left(\nu ,T\right)}{\partial T}=\frac{2\nu^{2}}{c^{2}}C_{E}(\nu ,T)=\frac{2\nu^{2}}{c^{2}}k_{B}{\left(\frac{h\nu }{k_{B}T}\right)}^{2}\exp\left(\frac{h\nu }{k_{B}T}\right)/{\left[1-\exp\left(\frac{h\nu }{k_{B}T}\right)\right]}^{2}$$

Frequency and temperature are independent variables in *I_BB_*, and thus in C*_E_*.

Because *C_E_* is on a per atom basis, a dimensionless conversion factor is needed to relate Equation (31) to measurements of a specific solid. From [4] (Chapter 11):

(38) $$\kappa_{lat,rad}(T)=\frac{V_{\mathrm{unitcell}}\rho N_{a}}{MZ}\int_{0}^{\mathrm{cutoff}}\frac{1}{A(\nu ,T)}\frac{\nu^{2}}{2c^{2}}C_{E}(\nu ,T)d\nu$$

where *M* is the mass in the formula unit, *Z* is the number of formula units in the unit cell volume, and *N_a_* is Avogadro’s number. The conversion factor is a dimensionless geometrical constant, and so is placed outside the integral. By taking logarithmic derivatives of *κ*, the geometrical constant and the above proposed numerical correction for *I*_BB_ become irrelevant.

#### 6.1.2. Limitations and Meaning of the Basic Formula

The inverse of the absorption coefficient has been represented as a mean free path. Actually, *A* describes attenuation (e.g., [102]). From Equation (1):

(39) $$A=-\frac{1}{I}\frac{\partial I}{\partial z}$$

Inserting Equation (39) in Equation (31) yields:

(40) $$\kappa_{rad}(T)=-\int_{0}^{\infty }I\frac{\partial L}{\partial I}\frac{\partial I(\nu ,T)}{\partial T}d\nu =-\int_{0}^{\infty }\frac{\partial L}{\partial T}I(\nu ,T)d\nu =-\frac{\partial L}{\partial T}\int_{0}^{\infty }I(\nu ,T)d\nu$$

The factor ∂*L*/∂*T* was extracted from the integral because it is a bulk material property. The negative sign results from heat flowing down the thermal gradient. For cubic symmetry *L*^3^ = *V*. Combining Equations (32), (35) and (40) gives:

(41) $$\kappa_{lat,rad}(T)=-\frac{const.}{4}\frac{\partial L}{\partial T}\frac{2\pi^{4}}{15}\frac{k_{B}{}^{4}}{h^{3}c^{2}}T^{4}=const.\frac{V^{1/3}}{3}\alpha \frac{\sigma_{SB}}{4\pi }T^{4}$$

where the constant and division by 4 comes from Equation (38).

The simplicity of Equation (41) and its extreme *T* dependence stems from an overly idealized process. First, optically thick conditions at every frequency are unachievable, as is reaching either limit of the integral in Equation (31) and its variants. Second, electrical insulators are highly transparent in the near-IR, yet a universal formulation for *D*(*T*) is indicated by measurements. Third, the blackbody curve is an unachievable idealization. The inelastic interactions which produce heat necessarily release less than 100% of the energy describing the interaction. This idealization is tied to the harmonic approximation, which describes *C_E_* and provides energy levels that are evenly spaced (multiples of h*ν*). Even spacing is not observed. Instead, spacing of energy levels decreases with the multiplicity. Fourth, under ordinary temperatures, lattice modes are excited. Electronic transitions in the visible (e.g., of Fe^2+^ in olivine) are negligibly excited at some hundreds of Kelvins. Hence, the cutoff frequency in Equation (38) is essential for energy conservation.

However, and importantly, under optically thick conditions and high *T*, Equation (31) depicting diffusion of blackbody radiation returns Fourier’s law (i.e., the inverted LHS of (2)), as can be seen by inserting Equation (32) into Equation (40) and recognizing that per steradian also depicts parallel rays. This finding is consistent with Fourier’s visualization of heat flowing through matter.

Thermal radiation is produced by heating matter. Stefan’s experiments provided the *T*^4^ law because these approximate blackbody radiation by heating a metal or graphite coated metal which have nearly constant *A* to high temperature. The intensity function diffusing inside a solid differs from that of a blackbody due to *A* varying with ν (Figure 12). On this basis, Equation (32) holds under all circumstances, where *I* is defined by Equation (39) for a medium without internal interfaces. Thus, our recasting of Equation (31) above shows that *κ* is the product of a length-scale (*L* = *V*^1/3^), with the physical property (α), which describes the material’s thermal response at constant *P*, and also with an integral describing the total thermal radiation flux inside the space where the material resides.

### 6.2. Equations for κ vs. P of Large Samples and Comparison with Data

Because the flux depends on *T* only per Equation (32), taking the *P* derivative of Equation (40) yields:

(42) $$\frac{\partial \ln\kappa_{rad}(T)}{\partial P}=-\frac{\partial \ln V^{1/3}}{\partial P}-\frac{\partial \ln\alpha }{\partial P}=\frac{1}{3B_{T}}-\frac{1}{\alpha B_{T}}\frac{\partial \ln B_{T}}{\partial T}=\frac{1}{B_{T}}\left(\frac{1}{3}+\delta_{T}\right)$$

Constants are removed via the logarithmic approach. The two representations to the right were obtained from thermodynamic identities. The dimensionless Anderson–Grüneisen parameter (*δ_T_*) varies but is close to 7 (see tables in [75,76,77,78]). Due to variations in *δ_T_*, the data are nearly but not exactly proportional to compressibility, i.e., the trend in Figure 14b shows scatter.

**Figure 15:**
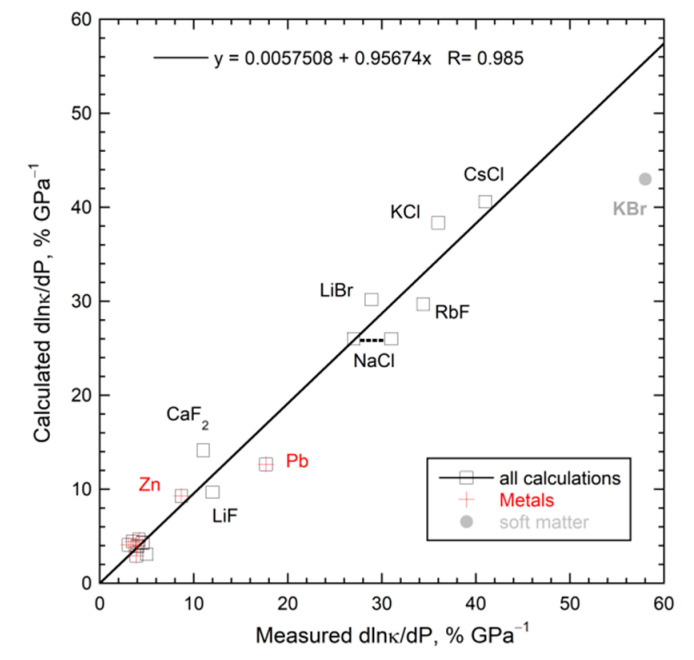
Calculations using (42) compared to measured transport data at pressure from Table 3. KBr exemplifies the soft, hydroscopic alkali halides (*B_T_* < 16 GPa). Reliable data on ∂*B*/∂*T* or δ_T_ were not found for Ni, Sn, or Gd.

Figure 15 shows that measurements agree with Equation (42) within substantial and variable uncertainties. The Anderson–Grüneisen parameter is uncertain by ~10% from comparisons of datasets including hard materials [78], and similarly for ∂ln*κ*/∂*P*. The pressure response of *κ*, modelled as the diffusion of radiation inside large samples, is described by thermodynamic properties because compression does not alter the properties of light. Equation (42) shows that Bridgmann’s parameter *g* exactly equals ⅓ + *δ_T_*, and furthermore underlies the exact relationship for lifetime depending on *P* (Equations (25) and (42) combined).

Table 3 and the figures above omitted NaClO_3_ due to hysteresis. From Equation (42), 51% GPa^−1^ is expected. Initial compression provided 80% GPa^−1^, whereas decompression provided 22% GPa^−1^ [103], which bracket the calculation. NaClO_3_ is hydroscopic, which may affect the determination of thermodynamic properties, as suggested by its inordinately large *δ_T_* = 14 [77]. Similarly, the hydration of the soft alkali halides is likely connected with disparities between (42), yielding ~40% GPa^−1^ and measurements yielding >58% GPa^−1^ [71].

The subscript “lat” was omitted from Equation (42) because Equation (40) holds generally. However only diffusion is described: co-exiting ballistic processes require a different formulation.

### 6.3. Approximate Equations for κ vs. T of Large Samples and Comparison with Data

Thermal conductivity is estimated from Equation (31) or Equation (38) here, following historical and modern calculations of heat capacity which approximate spectra as a delta function (Einstein), a triangular function with a cutoff frequency (Debye), or a boxcar alone or in combination with these historical approximations [104]. The boxcar shape better represents *C_P_* of insulators, whereas the triangular shape adequately describes metals (cf., [104,105]).

#### 6.3.1. One Mechanism of Heat Absorption

In modelling *κ*(*T*), a cut-on frequency of zero is used for the boxcar, which adds little uncertainty because absorption and intensities at very low frequencies are small. Simplified equations for a single mechanism thus involve one parameter (the cut-off frequency) and choosing a function for *A*(ν). Various power laws can be integrated [4] (Table 11.2). For *A,* proportional to ν^−1^, ν, and ν^2^, or constant *A*_0_, Equation (31) provides a peak in *κ* between 100 and 1000 K, such that the peak position depends strongly on the cutoff frequency [53] (Figure 11.8).

For the boxcar, (31) simplifies to:

(43) $$\kappa \left(T\right)=const.\left[\frac{24}{b^{3}}-\frac{e^{-b\nu }}{b^{3}}\left(24+24b\nu +12b^{2}\nu^{2}+4b^{3}\nu^{3}+b^{4}\nu^{4}\right)\right];\;b=\frac{1.44}{T}$$

where the subscript “cut-off” was dropped and wavenumbers are used. Maximum *κ* is proportional to *A*_0_. Data on ceramic sapphire up to ~1000 K are fit reasonably well [4] (Figure 11.5a).

For the triangular approximation using *A*~ν^2^, (31) simplifies to:

(44) $$\kappa \left(T\right)=const.\left[\frac{2}{b}-\frac{e^{-b\nu }}{b}\left(2+2b\nu +b^{2}\nu^{2}\right)\right];\;b=\frac{1.44}{T}$$

Equation (44) represents cryogenic data on several nearly pure metals [4] (Figures 11.5c and 11.9). However, metals at high temperatures and alloys need three parameters, as follows:

#### 6.3.2. Multiple Mechanisms and Complex Absorption Spectra

To allow for additional mechanisms that are associated with higher frequencies (e.g., overtones in electrical insulators), as well as for complex variations in *A* vs. ν, as in metals (Figure 12) which result from a continuum of transitions, the integral representing the fundamentals at low frequencies (e.g., (43) or (44)) must be summed with other integrals. Each integral represents one mechanism and involves different absorption coefficients and different frequency limits. For overtones (or impurity modes, as in Si), a boxcar beginning with ν = 0 is suitable due to their extreme difference in *A* values (about 1000-fold), which makes the overtone contribution negligible at low ν, in comparison.

For multiple processes, the contributions of the various integrals must be weighted by their heat capacities. Because non-dimensionalized data, *κ*(*T*)/*κ*_max_, are fit, the prefactor associated with integrating over the fundamental vibrations is inconsequential. However, the ratio of the prefactors for the overtone and the fundamental integrals is important to the fitting. This ratio combines the ratio of absorption coefficients with the ratio of weighting factors and obscures both. Thus, accounting for two mechanisms (or a wide range of frequencies) involves three parameters.

**Figure 16:**
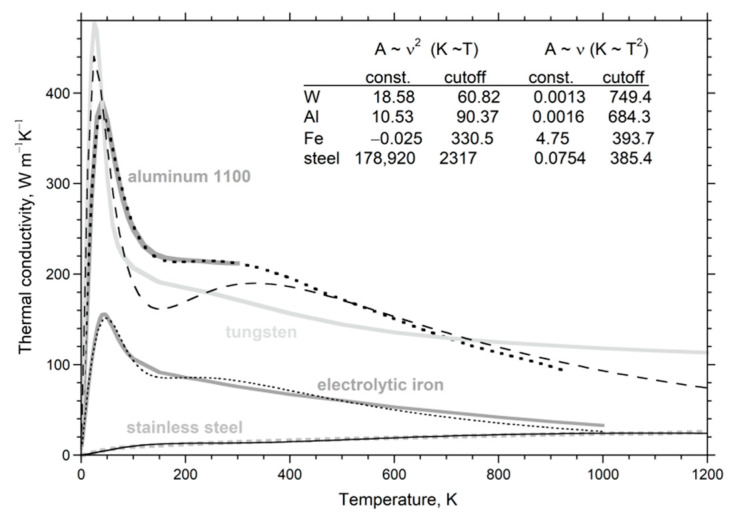
Fits to various metals using the radiative diffusion model with two mechanisms. We assumed *A*~*ν*^2^ for the infrared region and *A*~*ν* for the near-IR. Parameters are listed in the inserted table. If *κ* had been non-dimensionalized, only 3 parameters would be needed. Fits are in black. Data (grey) are from Hust and Lackford [24], except for Al from Bradley and Radebaugh [106]. Al-1100 is an alloy with about 1% impurities, usually Si or Fe. Tungsten is sintered, with non-negligible porosity. Modified after Figure 11.10 in Hofmeister [4], with permission from Elsevier.

We focus on metals which have the most accurate data on *κ*(*T*). The fit for iron (Figure 16) is affected by its Curie transition, causing one of the prefactors to be negative. Fits for Al and W involve much smaller prefactors for the contribution of the near-IR region, similar to the behavior of insulators. The thermal diffusivity of tungsten behaves much like the insulators (Figure 4). Steel has a large contribution at high *ν*, consistent with this alloy having many different cations and vibrations involving these impurities. Like steel, glasses are disordered, and also have *κ* which increases with *T* at high *T*.

The model for radiative diffusion produces *κ* (or *D*) that can either increase or decrease with *T*, depending on its absorption characteristics. This result is in accord with data in Figure 4, Table 1, and Equation (23) which represent diverse solids above 298 K with large *L*. Because the fitting formulae are based on integrals, the detailed dependence of *A* on *T* and ν is not essential, allowing model validity to be established via rough approximations of spectra. Data on *A*(*T*,*ν*) can be incorporated into the model for improved accuracy and predictive capability.

## 7. Conclusions

Heat flows in and out of solids, unaccompanied by the flow of mass. Inside a solid, heat flows down the thermal gradient, which is one-dimensional for the Cartesian systems largely utilized in experiments. The equivalence of light to heat from theory and spectroscopic experiments points to the mechanistic origin of heat flow in solids being the diffusion of radiation. Light is not affected by compression or expansion, although changes in temperature affect the thermal distribution of light frequencies. Planck’s blackbody curve describes an idealized distribution, which cannot be realized. Inside a solid, this distribution is instead defined by its absorption coefficient. In detail, this material property, *A*(*ν*,*T*), defines the length-scale, which produces optically thick conditions that are essential for diffusion. By relating intensity in the interior to attenuation (i.e., using the definition for *A*), we show that Fourier’s equation is identical to the independently derived formula describing diffusion of radiation.

Accurate data on thermal diffusivity as a function of sample length and temperature, as well as available and reliable data on thermal conductivity as a function of pressure near ambient temperature, are consistent with the diffusion of low frequency radiation (i.e., the infrared region) down the thermal gradient. As temperature increases, higher frequency light increasingly participates, as dictated by various transitions in the solid, which can be continuous (as in metals) or discrete (as in insulators). Applying our recast formula for diffusion of radiation to these data leads to the following findings:

- All solids respond to heat in the same manner, as demonstrated by simple approximations for *A*(*ν*) reproducing the known shape for *κ*(*T*) which describes metals, semi-conductors, and electrical insulators with diverse chemical compositions and crystallographic structures, as well as disordered materials (alloys and glasses).
- The exact temperature dependence of *κ* for any given material depends primarily on the frequency dependence of its absorption coefficient and secondarily on its temperature dependence. This material property, *A*, embodies how the substance interacts with light.
- The logarithmic pressure dependence of specific heat equals the negative of the linear compressibility (26). This new thermodynamic formula does not stem from Maxwell’s relations. This relationship shows that matter loses heat-energy during compression: i.e., squeezing reduces the space that a mass occupies but does not alter the amount of light flowing through that space. Linear, not volumetric compressibility, is relevant because heat flows in one direction, namely down the thermal gradient.
- The logarithmic pressure response of *κ* consists of two terms that sum. One term is exactly linear compressibility, which reflects loss of heat-energy as described in the preceding point. The other much larger term, ∂lnα/∂*P*, which is related to other equation-of-state parameters, describes how the space that a mass occupies depends on both temperature and pressure.

## Figures and Tables

**Table 2:** Dependence of thermal diffusivity at 298 K on thickness.

Sample	*D*_∞_	*b*	*R*	Sample	*D*_∞_	*b*	*R*
	mm^2^ s^−1^	mm^−1^		–	mm^2^ s^−1^	mm^−1^	–
MgO	18.867	1.8394	0.94	Ag	184.73	0.61894	0.97
Al_2_O_3_	13.738	1.5459	0.99	Cu	127.89	0.5872	0.99
Quartz ||c	6.7415	2.7419	0.88	Al	111.13	0.52831	0.99
Quartz ⊥c	3.963	2.7381	0.94	Si	109.02	0.64621	0.99
YSZ	0.73397	7.5016	0.96	Brass 260	43.764	0.85614	0.99
–	–	–	–	Pb	25.966	1.3854	0.99
–	–	–	–	Fe	23.654	1.8109	0.90
Ge124 glass ^1^	0.87352	11.111	0.68 ^1^	Steel ^2^	3.4556 ^2^	4.164 ^2^	0.97

^1^ Parameter *b* for glass is poorly constrained without data for *L* < 0.16 mm (samples fractured). ^2^ Three steels, from short to long, are 301 gasket material (2 points; this work), 310 [48], and SRM-1461 [39]. All are austenitic (non-magnetic) with similar compositions and should have roughly similar *D* at any given *T* and *L*. The parameters are also uncertain due to steel samples being hard and difficult to thin by grinding.

**Table 3:** Pressure dependence of thermal conductivity at ~298 K for *L* ~mm plus relevant thermodynamic data and lifetimes calculated from transport measurements.

Phase	*P*_max_ GPa	∂ln*κ*/∂P %GPa^−1^	∂ln*c_P_*/∂P %GPa^−1^	Ref. ^1^	*B*_T_^3,4,5^ GPa	∂*B_T_*/∂*T* GPaK^−1^	α μK^−1^	γ_th_	∂ln*τ*/∂*P* %GPa^−1^
Al	2.5	4.2	−1.8	[55]	72	−0.015	69.3	2.22	−5.5
Ag	2.5	4.0	−0.44 calc	[56]	103	−0.0215	56.7	2.34	−4.1
Au	2.5	3.9	−0.25 calc	[56]	163	−0.031	42.6	2.90	−3.9
Fe	1.6	3.5	−0.16 calc	[57]	163	−0.04	35.4	1.7	−3.5
Ni	1.6	4.4	−0.33	[57,58]	190.5		40.2	1.8	−4.6
Cu	2.5	3.1	−0.32	[59]	137.4	−0.036	49.5	2.02	−3.3
Zn	2	8.7	−0.67 calc	[60]	59.8	−0.0189	60.6	1.94	−8.8
Sn	1.1	13.4 avrg.	−0.56 calc	[51]	57		63.4	2.25	−13.4
Pb	1.1	17.7	−0.58 calc	[51]	43.2	−0.0192	86.7	2.65	−17.5
Gd	2.5	22	−2.0 calc	[61]	37			2.4	−23
AuCu disordered	1.6	5.6	−0.87	[62]	139		46.05	2.4	−6.2
AuCu_3_ ordered	1.4	5.9	−0.4	[62]	138		47.4	2.2	−6.1
AuCu_3_ disordered	1.9	3.2	~−0.4	[62]	138		47.4	2.2	−3.4
Si	1	4.0	−0.35 calc	[63]	100	(δ_T_ = 3.7) ^6^	7.8	0.16	−4.0
MgO	1.2	5.0	−0.4	[64]	160.2	−0.023	31.2	1.54	−5.2
MgO ceramic	5	4.7	–	[52]	–	–	–	–	–
Mg_1.8_Fe_0.2_SiO_4_	8.3	4.6 avrg.	−0.10 calc	[54] ^2^	128	−0.018	27.2	1.31	−3.8
Mg_1.8_Fe_0.2_SiO_4_	4.8	~4 optical		[65]	–	–	–	–	–
Garnet, natural	8.3	4.6	−0.04 calc	[54] ^2^	172	−0.029	23.6	1.5	−4.5
SiO_2_ glass	1	−4	+0.1	[66]	37	+0.016	1.5	0.5	5
SiO_2_ glass	9	−3.7	−2.7 calc	[53]	–	–	–	–	1.9
CaF_2_	1	11	−0.6	[67]	82.2	−0.0175	18.85	1.74	−11
LiF	1	12	−0.4	[67]	64.7	−0.377	97.8	1.60	−12
RbF	2	34.4	–	[68]	24	−0.122	95	1.4	–
NaCl	2	31	−1.75 calc	[69]	23.8	−0.016	118	1.58	−31
NaCl	1.7	27 optical	–	[70]	–	–	–	–	–
KCl	1.9	36	−2.35	[71]	17.3	−0.012	110	1.44	−36
CsCl	1.7	41	−4.0 calc	[69]	17.6	−0.147	144	1.99	−43
LiBr	2	28.9	−1.93 calc	[68]	24.3	−0.19	147	1.4	−29.5
KBr	1.7	58	−3.3	[71]	14.4	−0.10	116.4	1.45	−59

^1^ The cited reference refers to the data to the left. For *c_P_*(*P*), both measurements and calculations (calc) from thermodynamic data were made (see Section 5.3 for details). For Gd, the calculation describes the lattice, but not the magnetic contribution. Optical = optical method on a small sample. Avrg. = directionally averaged value. ^2^ Runs began at 2 GPa. Tabulated values of *κ* [54] are described by a linear fit with pressure, which was used to determine the derivative listed. For olivine, the derivative reported by [54] is lower, because an exponential function was used to match previous 1 atm data on thicker samples, which is not supported due to possible effects of *L*. ^3^ Unless noted otherwise, thermodynamic data were taken from the listed references and compilations. For metals and Si, bulk moduli were derived from B_T_ = B_S_/(1 + αγ_th_*T*), based on ultrasonic _BS_ values compiled by Raju et al. [72] and using γ_th_ = αB_S_/ρ*c_P_*, and/or compiled data on the web [73]. Much of the latter trace to the ~1970 volumes by Touloukian and collaborators, e.g., in [74]. For insulators, data are from several compilations [75]. Salt data compiled in [76], and checked against [77], were used for ∂*B_T_*/∂*T* or the Anderson–Grüneisen parameter. Both are uncertain, particularly the latter [78], being a lumped parameter (Section 5.3). ^4^ Thermodynamic data on fused silica glass compiled in [43]. ^5^ Thermodynamic data from [79]. ^6^ Anderson–Grüneisen parameter from [80].

## Data Availability

New and previously published data are contained within the figures and tables of this study. See http://epsc.wustl.edu/~hofmeist/thermal_data/ for previous data on thermal diffusivity and tabulations of data used to make the figures in the present study.
